# Heterocellular Coupling Between Amacrine Cells and Ganglion Cells

**DOI:** 10.3389/fncir.2018.00090

**Published:** 2018-11-14

**Authors:** Robert E. Marc, Crystal Lynn Sigulinsky, Rebecca L. Pfeiffer, Daniel Emrich, James Russell Anderson, Bryan William Jones

**Affiliations:** Moran Eye Center, Department of Ophthalmology and Visual Sciences, The University of Utah, Salt Lake City, UT, United States

**Keywords:** amacrine cell, ganglion cell, gap junction, GABA, retina, neural circuitry, transmission electron microscopy, computational molecular phenotyping

## Abstract

All *superclasses* of retinal neurons, including bipolar cells (BCs), amacrine cells (ACs) and ganglion cells (GCs), display gap junctional coupling. However, coupling varies extensively by *class*. Heterocellular AC coupling is common in many mammalian GC classes. Yet, the topology and functions of coupling networks remains largely undefined. GCs are the least frequent superclass in the inner plexiform layer and the gap junctions mediating GC-to-AC coupling (GC::AC) are sparsely arrayed amidst large cohorts of homocellular AC::AC, BC::BC, GC::GC and heterocellular AC::BC gap junctions. Here, we report quantitative coupling for identified GCs in retinal connectome 1 (RC1), a high resolution (2 nm) transmission electron microscopy-based volume of rabbit retina. These reveal that most GC gap junctions in RC1 are *suboptical*. GC classes lack direct cross-class homocellular coupling with other GCs, despite opportunities via direct membrane contact, while OFF alpha GCs and transient ON directionally selective (DS) GCs are strongly coupled to distinct AC cohorts. Integrated small molecule immunocytochemistry identifies these as GABAergic ACs (γ+ ACs). Multi-hop synaptic queries of RC1 connectome further profile these coupled γ+ ACs. Notably, OFF alpha GCs couple to OFF γ+ ACs and transient ON DS GCs couple to ON γ+ ACs, including a large interstitial amacrine cell, revealing matched ON/OFF photic drive polarities within coupled networks. Furthermore, BC input to these γ+ ACs is tightly matched to the GCs with which they couple. Evaluation of the coupled versus inhibitory targets of the γ+ ACs reveals that in both ON and OFF coupled GC networks these ACs are presynaptic to GC classes that are different than the classes with which they couple. These heterocellular coupling patterns provide a potential mechanism for an excited GC to indirectly inhibit nearby GCs of different classes. Similarly, coupled γ+ ACs engaged in feedback networks can leverage the additional gain of BC synapses in shaping the signaling of downstream targets based on their own selective coupling with GCs. A consequence of coupling is intercellular fluxes of small molecules. GC::AC coupling involves primarily γ+ cells, likely resulting in GABA diffusion into GCs. Surveying GABA signatures in the GC layer across diverse species suggests the majority of vertebrate retinas engage in GC::γ+ AC coupling.

## Introduction

Retinal ganglion cells (GCs) are the signal outflow cells of the vertebrate retina: a network layer that integrates bipolar cell (BC) and amacrine cell signals and passes them to CNS targets. Like BCs, most GCs are part of a unidirectional synaptic chain, not evidencing any direct feedback to the preceding input stage. However, early physiological studies established the ability of a GC to excite amacrine cells, other GCs and even itself (Matsumoto, 1975; Marchiafava, 1976; Marchiafava and Torre, 1977; Mastronarde, 1983; Sakai and Naka, 1988; Sakai and Naka, 1990). This excitation was always sign-conserving and occurred with short latency, yet electrical synaptic transmission was often dismissed due to a lack of anatomical evidence, in stark contrast to many other retinal neurons (Vaney, 1994). Later, intracellular biotinylated tracer injection studies (Vaney, 1991, 2002; Vaney and Weiler, 2000) showed tracer diffusion patterns between ganglion and amacrine cells that were interpreted as coupling mediated by gap junctions (e.g., Bloomfield and Xin, 1997; Massey, 2008), and more recently confirmed with gap junction protein knockout mice (e.g., Schubert et al., 2005a,b; Pan et al., 2010).

Gap junctions are intercellular channels that mediate the flux of small molecules and ions and, therefore, are the anatomical basis for electrical synaptic transmission in the nervous system. Like chemical synapses, gap junctions are extremely diverse structures mediating intercellular signaling. The primary proteins of gap junctions are drawn from a large family of connexins with four transmembrane spanning domains, cytosolic domains that usually (though not always) provide predominantly homotypic or bihomotypic binding even if the junctions are heteromeric (Li et al., 2008; Rash et al., 2013), and intracellular domains that mediate recognition and binding of other gap junction proteins. In general, it is thought that the peak open conductance of a single connexon is principally related to its pore diameter (this is not always true) with complex modulation enabled by a range of mechanisms (Ek-Vitorin and Burt, 2013; Hervé and Derangeon, 2013) including connexin phosphorylation (Pereda et al., 2013; O’Brien, 2017), methanesulfonate-analog (taurine) binding (Locke et al., 2011), and many different adapter protein interactions (e.g., Zou et al., 2017). Light-induced changes in gap junctions are currently understood to modify the open conductance of a connexon through these mechanisms, but will not change the presence or absence of gap junctions at contact sites with coupling partners. That said, photopic or scotopic changes may alter gap junctional sizes.

Modes of coupling in the retina can be grouped into broad categories such as homocellular (coupling between the same “types” of cells) and heterocellular (coupling between different cell types). But what do we mean by “type” in the context of retina? Our terminology is based on computational classification theory where a *class* is the ultimate level of granularity (Marc and Jones, 2002). In this terminology, mammalian rod photoreceptors, blue cones, rod BCs, and AII amacrine cells, are all classes. In contrast, the categories of photoreceptors, bipolar, amacrine and GCs are all *superclasses*, as they contain collections of classes or larger intermediate groups often defined *ad hoc* (see Supplementary Table S1). So what we really mean by heterocellular coupling is that it occurs between superclasses with clearly different morphologies, such as between AII amacrine cells and ON cone BCs. Homocellular coupling occurs within classes or between intermediate groups with the same morphology. Thus CBb3n::CBb4 coupling, where :: denotes the presence of gap junctions between the pair, is homocellular (between BCs) but is cross-class coupling engaging two different BC classes (Table 1; also see Mills, 2001). GCs are unique among retinal cells in favoring heterocellular over homocellular coupling. While sparse ultrastructure studies support in-class homocellular coupling for some GC classes (e.g., Hidaka et al., 2004), tracer coupling surveys (Bloomfield and Xin, 1997; Völgyi et al., 2009; Pan et al., 2010) of many GC classes suggests that most participate in heterocellular coupling with amacrine cells. In-class homocellular coupling, appears rarely, although it is impossible to distinguish between direct GC::GC coupling and indirect GC::AC::GC coupling when the tracer-labeled cohort includes both amacrine and GCs. Here, we show that specific GCs in the retina exhibit common rules for heterocellular coupling with amacrine cells, ranging from none to extensive. We have yet to identify instances of GC in-class homocellular coupling and have no proven cross-class homocellular coupling.

**Table 1:** Patterns of retinal coupling.

	Homocellular		Heterocellular	
Group	In-class	Cross-class	Cross-superclass	Partner
Rods	+	**∅**	+	Cones
HCs	+	**∅**	**∅**	
AI AC	+	**∅**	**∅**	
AII AC	+	**∅**	+	CBb BC
CBa BC	+	+	**∅**	
CBb BC	+	+	+	AII AC
RB	**∅**	**∅**	**∅**	
GC	**∅**	**∅**	+	γ ACs

While we know quite a bit about the general patterns of GC heterocellular coupling from tracer coupling studies, the network topology for the specific cell class partnerships involved and significance of coupling between the cell classes is elusive. Heterocellular coupling with amacrine cells subserves a circuit for synchronous GC firing (Brivanlou et al., 1998; Völgyi et al., 2013a), which may contribute to encoding aspects of the visual scene, such as direction (Meister and Berry, 1999; Ackert et al., 2006; Schwartz et al., 2007). There has also been discussion about whether coupling leads to maladaptive receptive field center expansion that would depress spatial resolution (Massey, 2008). However, two anatomical tools can assess the extent of coupling, enable precise definition of the partners and lead to more refined models of function: computational molecular phenotyping (CMP) and connectomics. While physiological analyses will always be definitive arbiters of global network functionality, connectomics can resolve network topologies that physiology cannot (e.g., Lauritzen et al., 2016). CMP allows quantitative specification of the small molecule signatures of retinal neurons, especially GCs (Marc et al., 1995; Marc and Jones, 2002). Here, we simply asked: what is the network embedding (in the mathematical sense) of GC::AC motifs? The answer is that for two specific GC classes, transient ON (tON) directionally selective (DS) and OFF alpha, heterocellular coupling exclusively occurs with multiple classes of γ+ amacrine cells that enable diverse modes of network specificity depending on the topology of the coupled inhibitory network. For the tON DS GC network, excitation of the GC may lead directly to the inhibition of neighboring GCs of differing classes.

Diffusion of small molecules, such as dyes and biotinylated tracers, through gap junctions has long been used to identify coupling between retinal cells (Vaney, 1994). Glycine, a small metabolite, readily identifies ON cone BCs due to glycine diffusion through gap junctions with AII amacrine cells, as cone BCs neither synthesize nor transport it (Cohen and Sterling, 1986; Vaney et al., 1998; Haverkamp and Wassle, 2000; Deans et al., 2002; Petrides and Trexler, 2008). Other small molecules are also likely to diffuse through gap junctions and accumulate, such as GABA from the γ+ amacrine cells to which the tON DS GC and OFF alpha GC are coupled. We show that both cells contain intermediate levels of GABA. In mammals, many classes of GCs exhibit an intrinsic GABA signal superimposed on a classic high-glutamate, high-glutamine and low taurine GC signature, suggestive of heterocellular coupling with γ+ amacrine cells (Marc and Jones, 2002). We note that no known GABA transporters have been described in any GCs, much less in the adult rabbit retina (Hu et al., 1999), and there are no studies that definitively report GAD in the GCs (in contrast to the amacrine cells in the GC layer), though there are studies that report GAD mRNA in developing rat retina (Brecha et al., 1991; Dkhissi et al., 2001), no functional protein has yet been identified. It should also be pointed out that the presence of GABA in the GCs does not imply that they are themselves, inhibitory. That circumstance would depend upon GABA vesicular loaders being present at the GC terminals. Rather, we only hypothesize about GABA being present due to coupling of GCs to amacrine cells where that GABA derives. It should also be noted that GABA is a central carbon metabolite that can be utilized for a number of biosynthetic pathways. As we will show, that signal is not unique to mammals.

## Materials and Methods

### Samples

Over 40 years our laboratory has collected retinal samples from over 50 vertebrate species spanning all classes. All euthanasia methods followed institutionally approved procedures, some of which changed over the years IACUC oversight evolved. Aquatic vertebrates were euthanized via cervical transection and double pithing (pre-1995) or sedated in 0.2% methanesulfonate prior to cervical transection (post-1995). Reptiles were similarly euthanized by cervical transection and double-pithing (pre-1995) or IP injection with 10% urethane followed by cervical transection. Mammals were euthanized by urethane overdose and thoracotomy (rabbits) or decapitation (pre-2014, mice), deep isoflurane anesthesia and thoracotomy or decapitation (2015); or Beuthanasia^®^ euthanasia and thoracotomy (rabbits, post-2015). The basic fixation method for all of them has been the same, as summarized in Marc (1999b): 250 mM glutaraldehyde, 1320 mM formaldehyde in either cacodylate or phosphate 0.1 M buffer pH 7.4, 3% sucrose, 1% MgSO_4_ or 1% CaCl_2_. All tissues were embedded in Eponate resins (Marc et al., 1978), serially sectioned at 100–250 nm onto array slides, probed for small molecules (Marc et al., 1998), visualized by quantitative silver-immunogold detection (Marc and Jones, 2002), and imaged as described below. Some retinas were incubated for 10 min in either teleost saline (Marc et al., 1995) or Ames medium (Marc, 1999a,b) containing 5 mM 1-amino-4-guanidobutane (AGB) and either 1 mM NMDA or 0.05 mM kainic acid for excitation mapping of retinal GCs.

### Immunocytochemistry

For the purposes of this paper, data from ≈20 years of post-embedding immunocytochemistry were analyzed and summarized. The same protocols and antibodies were used for all analyses. It is important to note that post-embedding immunocytochemistry for glutaraldehyde-trapped amines or imines is idempotent: once the sample is fixed and embedded, no detectable changes in immunoreactivity occur, even over decades. Indeed, tissues deriving from multiple species fixed in mixed glutaraldehydes and plastic embedded over 1980–1990 and published (Marc et al., 1990; Mills and Massey, 1992; Kalloniatis et al., 1996; etc…) have been directly compared with blocs of the same species (e.g., goldfish, rabbit, human, primate etc.) fixed in the past few years. They are indistinguishable. A good reference for this is Jones et al. (2003) where blocs of ≈30 individual transgenic rats had been prepared in the 1980s by Matthew LaVail. Rat retinas prepared post-2000 for this paper showed the same strength of GABA signals as blocs prepared in the 1980s. Signals were indistinguishable, and there is no published evidence showing any signal decline in resin embedded samples.

The key marker for heterocellular coupling is 4-aminobutyrate (GABA) detected in post-embedding immunocytochemistry (Marc, 1999b) using YY100R IgG (RRID:AB_2532061) from Signature Immunologics Inc. (Torrey, UT, United States). Additional channels for cell classification (Marc et al., 1995; Anderson et al., 2009, 2011b) targeted AGB (B100R, RRID:AB_2532053), glutamate (E100R, RRID:AB_2532055), aspartate (D100R, RRID:AB_2341093), glycine (G100R, RRID:AB_2532057), glutamine (Q100R, RRID:AB_2532059), and taurine (TT100R, RRID:AB_2532060) from Signature Immunologics Inc. For ease of notation, the Greek nomenclature for amino acids is used: GABA (γ), Glutamate (E), Glutamine (Q), Aspartate (D), Glycine (G), and Taurine (τ). AGB is denoted with (B). The activity tracer 1-amino-4-guanidobutane (AGB) is used to map both endogenous and exogenous ligand-driven glutamatergic signaling in single cells. Guanidinium cations are permeable to a wide variety of non-selective cation channels. The Guanidinium analog, AGB has demonstrated the same non-selective cation channel permeability to that seen by guanidinium (Yoshikami, 1981; Qwik, 1985; Kuzirian et al., 1986) and can be utilized as channel permeant markers by selectively activating glutamate receptors (Marc, 1999b; Marc and Jones, 2002), and allowing AGB to diffuse in along a concentration gradient. In essence, the tissue is incubated in a high concentration of AGB, which enters the cell through cation channels when the cell is activated. In the case of RC1, a flicker photopic light was used to drive neuronal classes allowing AGB entry via cation channel opening in response to glutamate receptor activation in neuronal classes (Marc, 1999a,b; Marc and Jones, 2002; Marc et al., 2005; Anderson et al., 2011b). All IgGs were detected with silver-intensified 1.4 nm gold granules coupled to goat anti-rabbit IgGs (Nanoprobes, Yaphank, NY, SKU 2300), imaged (8-bit monochrome 1388 pixel × 1036 pixel line frames) in large mosaic arrays with a 40× oil planapochromatic objective (NA 1.4) on a 100 × 100 Märzhäuser stage and Z-controllers with a QImaging Retiga camera, Objective Imaging OASIS controllers, and Surveyor scanning software (Anderson et al., 2011b; Lauritzen et al., 2016).

Raw signal is used to describe the original image acquired following staining without any image processing. Density mapped images are obtained from light microscopy of the silver intensified antibody labeled images. In these images, darkness of a region indicates a higher density of antibody labeling. Intensity mapped images are the inverted image of density mapped images, we invert these images to better facilitate the readers ability to interpret the small molecule mixtures within cells. Theme mapping is the assignment of a color to each cell class generated through k-means cluster analysis and overlaid in the same space as the original image to visualize which cells cluster together, and therefore have the same cell signature. Segmentation of cell classes using amino acid labeling was performed as previously described (Marc et al., 1995; Marc and Jones, 2002). In brief, IgG labeled sections were co-registered and clustered as N-dimensional images using *k*-means. Each separable cluster is made up of a distinct signature of concentrations of multiple amino acids unique to that cell class. The clustering results were then remapped in the same *x*–*y* dimensions as the original IgG image. This graphical representation of the cell classes is termed a theme map. Using the theme map as a mask, the underlying histograms can be evaluated for each cell class, where the histogram demonstrates the approximate log concentration of small molecule within the cell. For a more comprehensive review of these methods see Marc and Jones (2002). Image analysis, histogram thresholding, object counts and spacing measures were performed using ImageJ 2.0.0-rc-43/1.51w (Rueden et al., 2017) in the FIJI Platform (Schindelin et al., 2012) and Photoshop CS6 (Lauritzen et al., 2016).

### Connectomics in Rabbit Retinal Volume RC1

Connectome assembly and analysis of volume RC1 has been previously described (Anderson et al., 2009, 2011a,b; Lauritzen et al., 2012, 2016; Marc et al., 2013, 2014a) and only key concepts expanded here. RC1 is an open-access rabbit retina volume imaged by transmission electron microscopy (TEM) at 2 nm and includes 371 serial 70–90 nm thick sections, with six and twelve optical sections flanking the inner nuclear and ganglion, cell layers, respectively, containing small molecule signals and additional intercalated optical sections throughout (Anderson et al., 2011b). The retina was dissected from euthanized light-adapted female Dutch Belted rabbit (Oregon Rabbitry, OR) after 90 min (under 15% urethane anesthesia, IP) of photopic light square wave stimulation at 3Hz, 50% duty cycle, 100% contrast with a 3 yellow – 1 blue pulse sequence (Anderson et al., 2011b) with 13–16 mM intravitreal AGB. All protocols were in accord with Institutional Animal Care and Use protocols of the University of Utah, the ARVO Statement for the Use of Animals in Ophthalmic and Visual Research, and the Policies on the Use of Animals and Humans in Neuroscience Research of the Society for Neuroscience. Each retinal section was imaged as 1000–1100 tiles at 2.18 nm resolution in 16- and 8-bit versions, and as image pyramids of optimized tiles for web visualization with the Viking environment (Anderson et al., 2011a). Synapses and other intercellular relationships and intracellular structures were identified anatomically from TEM images and re-imaged at 0.27 nm resolution with goniometric tilt where necessary for validation. Neural networks in RC1 have been densely annotated with the Viking viewer (Lauritzen et al., 2016), reaching over 1.4 million annotations of 3D rendered volumetric neurons, processes, pre- and postsynaptic areas, locations in the volume with subnanometer precision (Jensen and Anastassiou, 1995), and explored via graph visualization of connectivity and 3D renderings as described previously. The volume contains ≈1.5 M annotations, 104 rod BCs, >145 classified, 24 unclassified, 10 classified partial arbors, 300 amacrine cells and 20 GC somas. This density of annotations belies the additional work required to validate, classify and scale. Each annotation is a size and location entity coupled to a full metadata log (Anderson et al., 2011a) and has been validated by at least two tracing specialists; many have been revisited 5–10 times, representing a total of 7 person-years of work. No current automated tracing tool makes fewer errors than a trained human annotator (even our own: Jagadeesh et al., 2013). Therefore, any time saved by automation is negated by the necessity for human cross-checking, validation and correction/re-annotation. Rendered neurons in RC1 were produced in Vikingplot (Anderson et al., 2011a,b) and VikingView (Lauritzen et al., 2016).

### Mining Coupled Ganglion Cell Networks

Candidate GC coupling networks in RC1 were visualized and annotated by identifying GABA-positive (γ+) GC somas and dendrites in Viking^1^ (RRID:SCR_005986) in the intercalated GABA channels and by searching the RC1 database for coupling connections using network graph tools and database queries. All resources are publicly accessible via Viking and a range of graph and query tools are available at connectomes.utah.edu. All cells in this article are numerically indexed to their locations, network associations, and shapes. The data shown in every TEM figure can be accessed via Viking with a library of ^∗^.xml bookmarks available at marclab.org/GCACcoupling. Each cell index number in the RC1 database can be entered into different software tools for analysis, visualizations, or queries: Viking, Network Viz, Structure Viz, Info Viz, Motif Viz (Viz tools are based on the GraphViz API^2^, developed by AT&T Research, RRID:SCR_002937), and VikingPlot developed by the Marclab; and VikingView developed by the University of Utah Scientific Computing and Imaging Institute. Further, Viking supports (1) network and cell morphology export into the graph visualization application Tulip^3^ developed by the University of Bordeaux, France; (2) cell morphology for import into Blender^4^ (RRID:SCR_008606); and (3) network queries for Microsoft SQL and Microsoft Excel with the Power Query add-in to use the Open Data Protocol (OData.org) to query connectivity features. More efficiently, we discover and classify coupling networks in Tulip with TulipPaths: a suite of regex (regular expression) based Python plug-ins for network queries^5^^,^^6^. Tulip networks can be directly exported from our connectome databases with a web query tool at connectomes.utah.edu and all data used in this article can be accessed via marclab.org/GCACcoupling.

### Statistics

Small molecule signal comparisons across groups were done by both *k*-means clustering and histogram analysis using PCI Geomatica (Toronto, Canada) and CellKit based on IDL (formerly ITT, now Harris Geospatial, Melbourne, FL, United States) as described in Marc and Jones (2002). Various parametric and non-parametric analyses of feature sets (e.g., gap junction numbers, sizes) and power analyses were performed in Statplus:mac Version v6^7^ (RRID:SCR_014635) and R^8^ (RRID:SCR_001905).

### Signatures

The signature hypothesis is the concept that each morphologically and functionally distinct cell would also possess distinct neurochemical compositions (Burnstock, 1976; Watt et al., 1984). We define the signature as quantitative differences in small molecule concentration mixtures as determined by k-means cluster analysis, indicating unique cell classes.

## Results

### Phylogeny of Heterocellular Ganglion Cell Coupling With GABAergic Amacrine Cells

Our analysis of two γ+ GC classes in connectome RC1 demonstrates a mechanism by which the small molecule GABA could accumulate in GCs: heterocellular coupling via numerous small gap junctions with sets of γ+ amacrine cells. Thus, GABA signals superimposed on a classic high-glutamate, high-glutamine, and low taurine GC signature, can in turn be used to screen vertebrates for possible heterocellular GC::AC coupling. Specifically, cells in the GC layer with GABA signal histograms matching those of conventional amacrine cells (1–10 mM) are classified as displaced amacrine cells and those with intermediate signals (0.1–1 mM) are classified as provisionally coupled GCs (see Marc and Jones, 2002 for calibrations). In many species, we are also able to correlate these intermediate GABA levels with classical high glutamate signals of GCs and distinctly large GC sizes (e.g., Marc and Jones, 2002). Using the marclab.org tissue database we reviewed 53 vertebrate species spanning all vertebrate (Supplementary Table S2) classes to assess the scope of potential coupling. Importantly, evidence of GC heterocellular coupling with GABAergic amacrine cells occurs in *every vertebrate class*, even if other markers of comparative function vary: e.g., Müller cell GABA transport (limited to Cyclostomes, Chondrichthyes, Mammals and advanced fossorial ectotherms such as snakes), horizontal cell GABA transport (limited to most bony ectotherms) and horizontal cell GABA immunoreactivity (dominant in bony ectotherms and variable in mammals). The only vertebrate class we can say appears to clearly lack evidence of heterocellular GC::AC coupling is Testudines: turtles.

**FIGURE 1:**
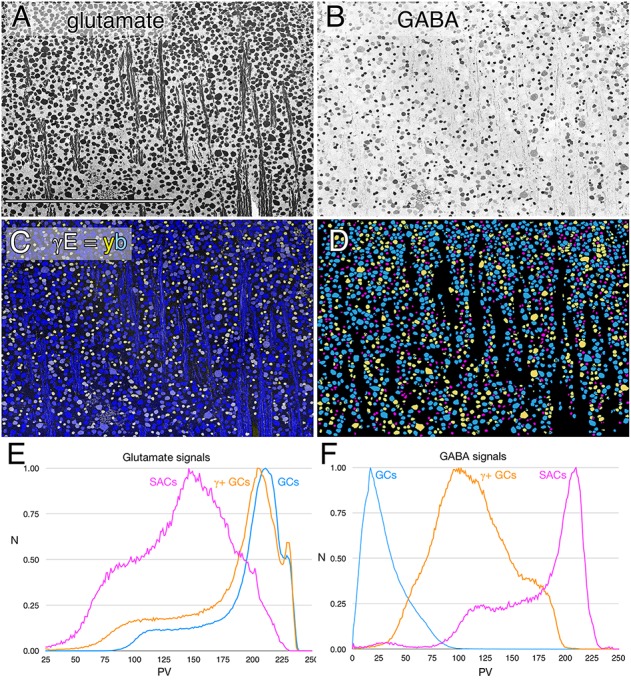
Glutamate and GABA colocalization in the rabbit ganglion cell layer; registered serial 200 nm horizontal sections through the plane of the ganglion cell layer with silver density visualization. **(A)** Raw glutamate signals of ≈2100 cells, density mapped; Scale, 0.5 mm. **(B)** Raw GABA signals of the same cells, density mapped. **(C)** Intensity mapped [inverse images of **A,B**)] registered channels with GABA (γ) signals encoded as a yellow (y) channel (R + G) and glutamate signals (E) as the blue (b) channel (γE = yb mapping) to create additive and quantitative concentration maps that reflect the free amine content of the cells. **(D)** Theme mapped data produced through GABA histogram segmentation. Magenta: high GABA content (5–10 mM) population containing mostly starburst amacrine cells and a few displaced amacrine cells. Yellow: medium GABA content population containing provisional γ+ ganlion cells (0.1–1 mM). Cyan: Ganglion cells with no measurable GABA content (<0.1 mM); small fragments represent portions of cross-sectioned cell dendrites. **(E)** Glutamate histograms of peak normalized pixel number (N) vs. pixel value (PV) for starburst amacrine cells (SACs), GABA-positive ganglion cells (γ+ GCs) and GABA-negative ganglion cells (GCs). The pixel value is the digital grayscale readout from the raw imagery, ranging from 0 to 255 and the peak normalized pixel number is the normalized maximum frequency of pixels in the image for a given pixel value. Pixel value reflects the quantitative amounts of small molecules which are log-linearly scaled with histogram pixel value representing an approximation of concentration (Marc et al., 1995). **(F)** GABA histograms of peak normalized pixel number (N) vs. PV for starburst amacrine cells (SACs), GABA-positive ganglion cells (γ+ GCs) and GABA-negative ganglion cells (GCs).

In every vertebrate class that shows a potential coupling profile, the GABA signal and GC types involved are diverse. Figure 1 shows the spectrum of GABA signals in the rabbit GC layer, just below the visual streak, obtained by registering the glutamate (Figure 1A) and GABA (Figure 1B) channels of 2385 cells in the GC layer. The signals in Figure 1B reveal that GABA levels range from undetectable in many cells to levels that nearly match those of conventional amacrine cells, starburst amacrine cells in particular. In between are a range of concentrations far lower than any GABAergic amacrine cell (Marc and Jones, 2002) but much higher than background. Our previous assessments of the selectivity of the YY100R anti-GABA IgG (Marc and Jones, 2002) and competition assay results are shown in Supplementary Table S3, and range from 10^4^ to 10^6^ log units in concentration. Thus, the intermediate values cannot be due to cross reactivity with any plausible alternate biomarkers (e.g., L-alanine, β-alanine, taurine, etc.), else they would have to be present at levels of 1–100 M (100 μM low signal range × 10^4^–10^6^ cross-reactivity), which is physically impossible. Glutamate concentrations seen in GABAergic neurons is over a log unit lower than levels of glutamate found in presumptive glutamatergic cells. This range of glutamate immunoreactivity in GABAergic neurons has been described before (Marc et al., 1990, 1995) and it is likely that all GABA cells have at least some detectable glutamate given that glutamate is a central carbon skeleton metabolite and is the direct precursor to GABA synthesis via glutamate decarboxylase (GAD).

The intermediate ranges of GABA signals are associated with GC soma sizes ranging from some of the largest to some of the smallest GCs (Figure 1C), and the GC layer is separable by either clustering or histogram thresholding (Marc et al., 1995) into pure glutamate signal GCs (uncoupled), γ+ GCs (provisionally coupled) and starburst and minor displaced amacrine cell cohorts (Figure 1D). Importantly, all γ+ GCs show glutamate signatures indistinguishable from γ- pure glutamate GCs (Figure 1E), while starburst and other displaced amacrine cells display much lower glutamate contents similar to γ+ amacrine cell signatures in various vertebrate species (Marc et al., 1995; Marc, 1999a). The amacrine cell cohort is unique in quantitative glutamate and GABA signatures, and soma size, while the γ+ GCs and γ- GCs are not discriminable in glutamate signatures, or soma size and are separable as an intermediate class only by the modest GABA signatures of γ+ GCs.

**FIGURE 2:**
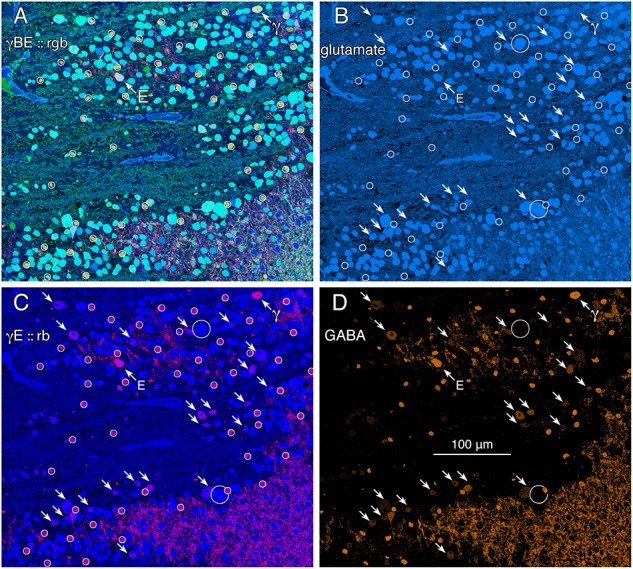
Glutamate and GABA colocalization in the goldfish ganglion cell layer; registered serial 200 nm sections with silver density visualization inverted (with a logical NOT) to an intensity display (Marc et al., 1995). **(A)** GABA (γ), AGB (B), and glutamate (E) signals assigned to the red (r), green (g), and blue (b) channels, respectively, creating an rgb image reflecting the combined small molecule signature. AGB permeation was activated *in vitro* with 50 μM kainic acid (KA) in the presence of 10 mM AGB in Hickman’s Teleost saline (Marc et al., 1995). This signature separates ON starburst amacrine cells (small circles) with a yellow signal mixture (high GABA and AGB, representing classic strong starburst amacrine cell KA responses) from cyan ganglion cells (high glutamate and AGB, representing strong ganglion cell responses to KA), light blue ganglion cells (high glutamate, low AGB, representing weak ganglion cell responses to KA), and deep blue spherical terminals of Mb ON cone bipolar cells (surrounded by a polygon), which lack ionotropic glutamate receptors. The northwest arrow labeled with the E symbol indicates a high glutamate content ganglion cell with modest GABA and high AGB signals. The northwest arrow labeled with the γ symbol indicates a low glutamate, high GABA, non-starburst amacrine cell. **(B)** Glutamate channel, intensity mapped as medium blue for visibility (R = 0, G ≈ 0.5B, B ≈ 0–240). Southeast arrows denote high glutamate ganglion cells that also have significant GABA signals. **(C)** GABA and glutamate channels mapped as γE :: rb, revealing γ+ ganglion cells as magenta cells. **(D)** GABA channel mapped as orange for visibility (R ≈ 0–240, G ≈ 0.5R, B = 0), clearly revealing weak GABA signals in a set of high glutamate ganglion cells. Scale, 100 μm.

GABA signals in GCs are not unique to mammals. Teleost fishes represent the Actinopterygii, a vertebrate class with ≈400 Mya divergence from class Sarcopterygii, while infraclass Teleostei is of even more modern origin (≈310 Mya) with a massive post-Mesozoic, early Cenozoic expansion (Friedman, 2010) contemporaneous with mammalian speciation. The emergence of the cyprinids (goldfish and zebrafish) is extremely modern with estimated peak speciations in the Miocene and even early Holocene (Dubut et al., 2012), postdating the divergence of anthropoid primates (Pozzi et al., 2014). Arguably, comparing mammals and teleosts is one of the most diverse spans that could be conceived in assessing a putative synapomorphy (specialization of a clade) such as coupling, with a last common ancestor in the Devonian. Figure 2 shows varying GABA signals exhibited by cells within the GC layer of the goldfish *Carassius auratus*. There are clearly different classes of GCs with GABA signals that are below the amacrine cell signal range (Figure 2, northwest arrow labeled with the E symbol and southeast arrows). As a control, the population of goldfish starburst amacrine cells (small circles) forms a single signature cohort with GABA levels much higher than provisionally coupled GCs. Their glutamate, GABA and kainate-activated AGB signals show that they form a distinct, monolithic, inseparable signature group that cannot be drawn from any other population, while presumably coupled and uncoupled GCs have much weaker (or no) GABA signals and diverse signatures (Figure 2).

### Ultrastructural Evidence of Heterocellular GC::AC Coupling

**FIGURE 3:**
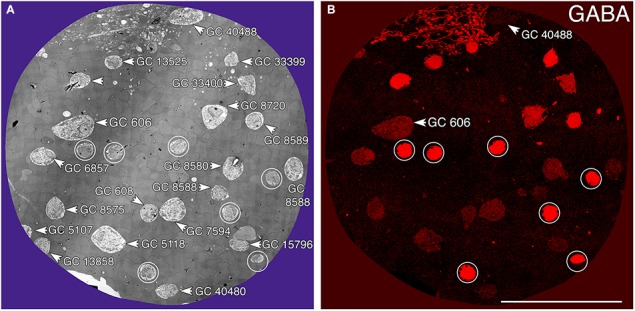
Ganglion cell - GABA colocalization in retinal connectome RC1. **(A)** Slice 371 TEM image displaying somas of 20 GCs (numbered) and 7 ON starburst ACs (circled). GC 606 is the largest GC soma in the volume with major and minor diameters of 34 and 19 μm. **(B)** Slice 371 GABA channel (Anderson et al., 2011b) with γ+ GC 606 and γ– GC 40488 labeled and starburst amacrine cells circled as in **(A)**. Scale, 100 μm.

Tracer coupling suggests widespread heterocellular GC::AC coupling in the vertebrate retina, and significant correlative evidence supports that view (Völgyi et al., 2013a). But what are these coupled amacrine cells and what networks do they comprise? What is the relationship between coupling and gap junction expression? This is where retinal connectomics can offer critical insights. To direct our tracing efforts, we took advantage of the integrated CMP in connectome RC1. Most amacrine cells utilize either GABA or glycine as a neurotransmitter, which likely diffuses through gap junctions into coupled GCs. Consistent with this, many GC classes exhibit a range of GABA signals, but well below that of conventional amacrine cells (Marc and Jones, 2002). The GC layer in rabbit retinal connectome RC1 contains the somas of 20 GCs and 7 ON starburst amacrine cells (Figure 3A). Several of the GCs show significant levels of GABA (Figure 3B), suggesting they may couple with γ+ amacrine cells. We have reconstructed the gap junction patterns of two major classes of γ+ GCs, the tON DS GC GC 606 and a length of dendrite within the inner plexiform layer of the OFF alpha ganglion cell GC 9787 (not shown in Figure 3 as the soma is not contained within the RC1 volume). We traced most of the connections of both of these cells in connectome RC1 and demonstrate that both are extensively coupled to unique sets of γ+ amacrine cells.

### GC 606

**FIGURE 4:**
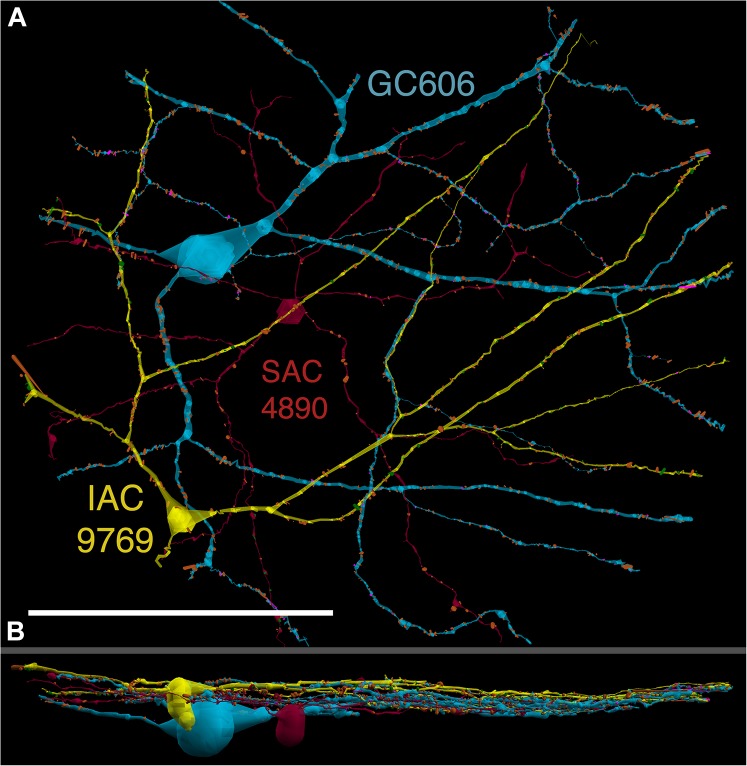
**(A)** XY projection of commingled GC 606 (light blue) and IAC 9769 (yellow) dendritic arbors just distal to the dendrites of starburst amacrine cell (SAC) 4890 (red) in the inner plexiform layer of connectome RC1. Computer generated three dimensional rendering of Viking annotations generated with VikingPlot. Small dots indicate the relative sizes (scaled by a factor of 2 for visualization) and locations of presynaptic specializations (green), PSDs (orange), gap junctions (magenta). **(B)** XZ projection demonstrating lamination of GC 606, SAC 4890, and IAC 9769. Scale, 100 μm.

GC 606 has a large, γ+, crescent-shaped soma with a maximum diameter of 35 μm positioned within the GC layer of connectome RC1 (Figure 3). Its GABA signal is strong, albeit at much lower concentrations than truly GABAergic amacrine cells such as ON starburst amacrine cells. Its dendritic arbor spans the entire RC1 volume, extending beyond its boundaries in all directions, and appearing to fully stratify within sublamina b of the inner plexiform layer, just distal of the ON starburst amacrine cell dendritic stratification within the inner plexiform layer (Figure 4). GC 606 is indisputably an ON GC. Its excitatory synaptic input exclusively arises from ON cone BCs. GC 606 heavily couples with at least two classes of γ+ amacrine cells, including an interstitial amacrine cell (IAC) consistent with the γ+ PA1 polyaxonal cell (Famiglietti, 1992; Wright and Vaney, 2004) with which it extensively co-stratifies (Figure 4). Due to this coupling, GC 606 cannot be an ON alpha GC (Hu and Bloomfield, 2003) nor a classic sustained ON directionally selective (DS) GC (Hoshi et al., 2011). Moreover, there are no starburst amacrine cell inputs, further supporting that it cannot be a classic sustained ON DS GC (Hoshi et al., 2011). The soma size, arborization level, γ+ coupling and lack of starburst inputs are all consistent with the classification of GC 606 as a tON DS GC, known to be tracer coupled to at least two classes of γ+ amacrine cells, one of which is clearly an IAC (Ackert et al., 2006, 2009; Hoshi et al., 2011; Massey, personal communication).

**FIGURE 5:**
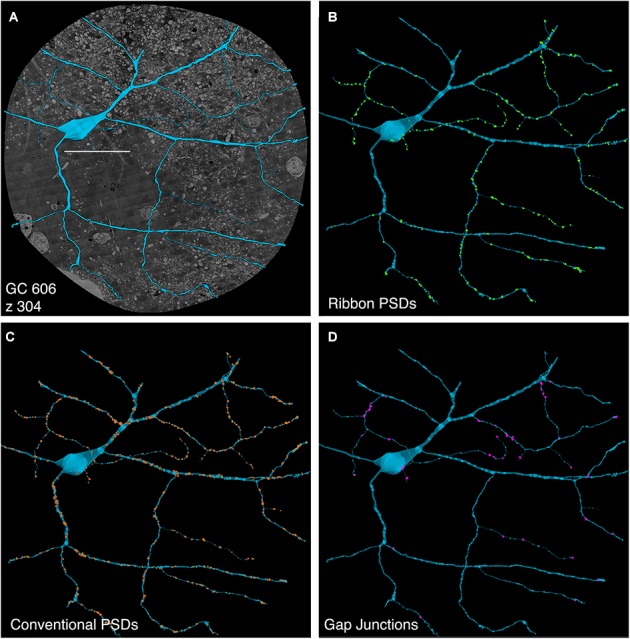
Computer rendering of GC 606 **(A)** superimposed on connectome slice z304 with separate displays of **(B)** excitatory ribbon synapse PSDs, **(C)** inhibitory conventional synapse PSDs and **(D)** 49 confirmed out of 228 identified gap junctions each displayed at 2× their true diameters. Most gap junctions are with partner GABA+ amacrine cells in Figures 9, 10. Scale, 100 μm.

The initial stage of characterizing a neuron in a connectome is defining its excitatory, inhibitory and coupling drive (Figure 5). The drive for GC 606 extracted by data queries from connectome RC1 is summarized in Table 2 for 1267 validated contacts. As in previous analyses of the inner plexiform layer (Marc and Liu, 2000), synaptic drive is dominated by inhibition with ≈3 inhibitory synapses per excitatory input and 5.5 μm^2^ of inhibitory PSD area per μm^2^ of ribbon PSD. By measuring dendrite lengths of representative cells from Hoshi et al. (2011), we estimate that GC 606 represents only 18% of the dendritic length of a complete tON DS GC. Thus, a complete tON DS GC should receive ≈1440 excitatory ribbon synapses driving ≈54 μm^2^ of PSD area; ≈4350 inhibitory conventional synapses driving ≈290 μm^2^ of PSD area; and make ≈1270 gap junctions summing to 35 μm^2^ of coupling area across its arbor (assuming no dramatic influence of eccentricity on the frequency of these interactions). However, this comprises only about 6% of the gap junction density in the inner plexiform layer (Marc et al., 2014a) and since many of the gap junctions are suboptical, tracing them by fluorescence imaging (even super-resolution methods) could be challenging.

**Table 2:** Contacts of GC 606.

Feature	*n*	Mean area μm^2^ ± 1SD	Area range μm^2^	606 total area μm^2^	GC total area μm^2^	Area/μm^2^
Ribbon synapse PSDs	259	0.038 ± 0.023	0.009–0.153	9.8	54	0.005
Inhibitory synapse PSDs	783	0.068 ± 0.034	0.067–0.335	53.6	294	0.030
Gap junctions	228	0.028 ± 0.017	0.004–0.100	6.4	35	0.004

**FIGURE 6:**
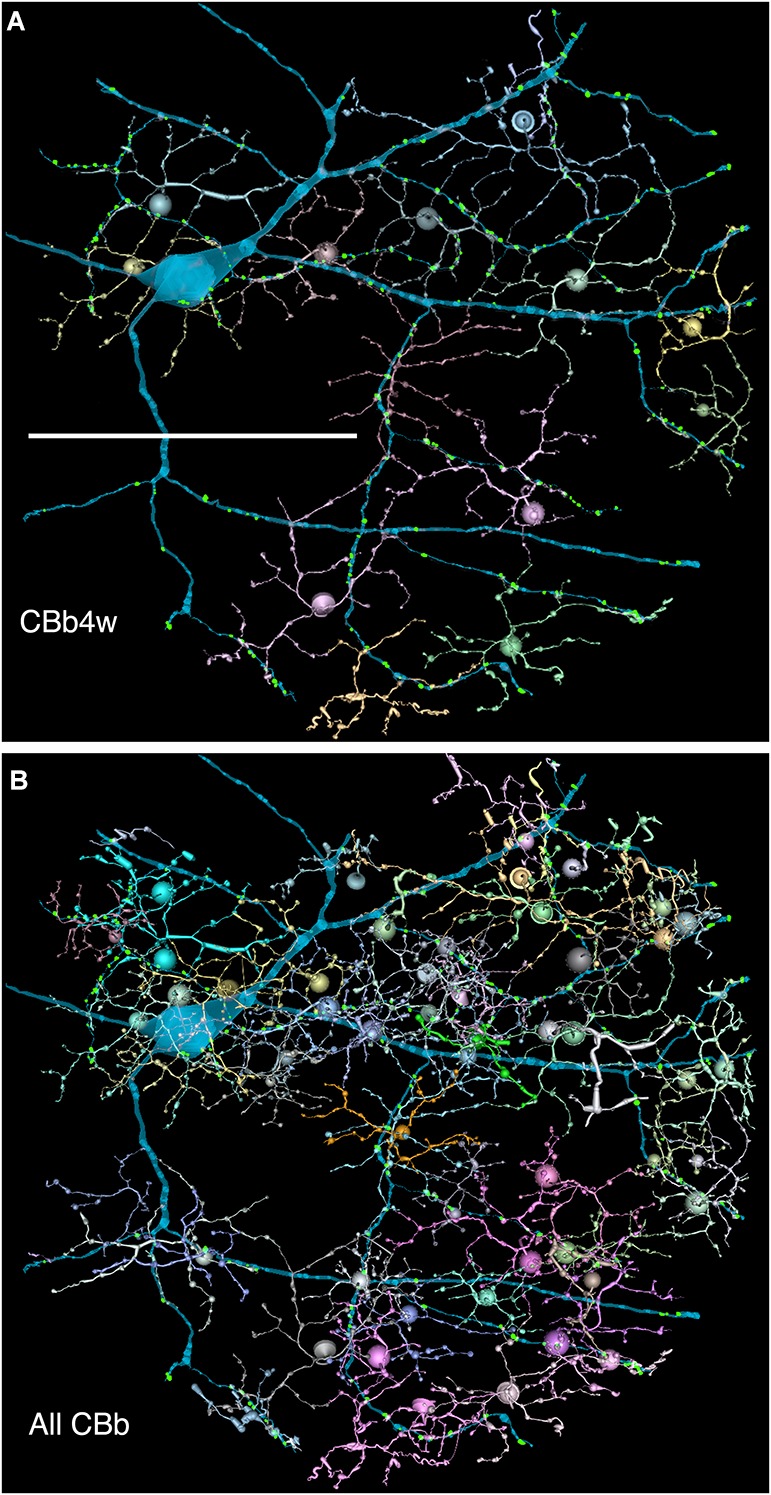
**(A,B)** Are combined VikingPlot and VikingView renderings. GC 606 and its bipolar cell input. **(A)** The dominant synaptic ribbon drive (58%) arises from a single class, CBb4w, a coupled homocellular network of ON cone bipolar cells. Each cell is colored individually. **(B)** The entire CBb input cohort to GC 606. Scale, 100 μm.

**FIGURE 7:**
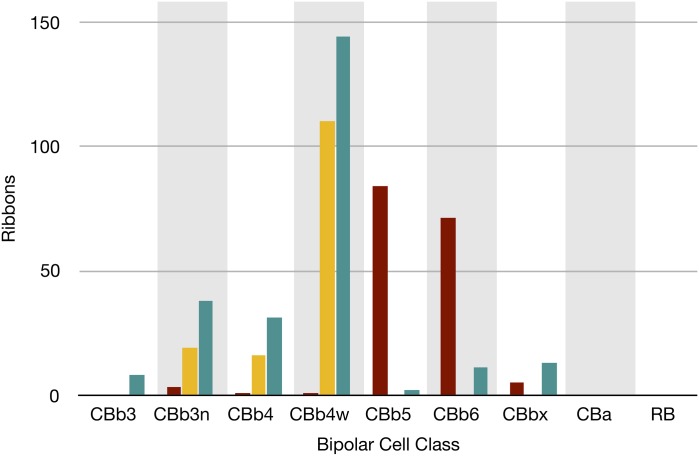
The classes of bipolar cell input to GC 606 (Cyan, *n* = 247), IAC 9769 (gold, *n* = 145) and all ON starburst amacrine cell (SAC) dendrites (red, *n* = 165) in RC1. Ordinate: number of synaptic ribbons from each class. Abscissa. All bipolar cell groups, including ON cone bipolar cell classes CBb3, CBb3n, CBb4, CBb4w, CBb5, CBb6, the aggregate OFF cone bipolar cell superclass (CBa) and the rod BC class. CBbx cells are ON cone bipolar cells from the volume margins with insufficient reconstruction to allow identification.

Excitation patterns are class-specific. GC 606 receives glutamatergic excitation exclusively from ON cone BCs as can be shown by querying the RC1 database with the TulipPaths plugin (see section “Materials and Methods”): e.g., query “CB.^∗^, ribbon, GC ON” which returns all the cone BC ribbon synapses onto specific ON GCs from identified BCs (Figures 6, 7). Of the 259 ribbon complexes that drive GC 606 in RC1, 54% originate from one class of BCs, CBb4w (Figure 6A), and over 99% of the input *excludes* CBb5 BCs, which represent the primary drivers of ON starburst amacrine cells (Figure 7). This is largely due to stratification. CBb5 BCs stratify just proximal to CBb4w BCs in the inner plexiform layer with only marginal overlap of their axonal arbors (Lauritzen et al., 2016). Likewise, the ON starburst amacrine cell 4890 stratifies just proximal to GC 606 (Figure 4), consistent with previous descriptions of tON DS GCs (Hoshi et al., 2011). As a reference, the highly coupled IAC 9769 extensively commingles with GC 606 and samples many of the same BCs with an even narrower preference spectrum dominated by CBb4w and effectively excluding CBb5 (Figure 7). In contrast, ON starburst amacrine cells contact a different profile with over 90% of their inputs deriving from CBb5 and CBb6, and less than 1% from CBb4w (Figure 7). While ON starburst amacrine cells make numerous synapses onto GC dendrites in the RC1 volume, they make no synapses onto either GC 606 or IAC 9769, nor do they receive any synapses from IAC 9769.

In addition to its extensive excitatory cone BC input, GC 606 also collects 783 conventional inhibitory chemical synapses from amacrine cells. Of those that are neurochemically identified, 33 have been mapped to definitive γ+ amacrine cells and only two to G+ amacrine cells as they traverse GABA or glycine reference slices (see Anderson et al., 2011b).

**FIGURE 8:**
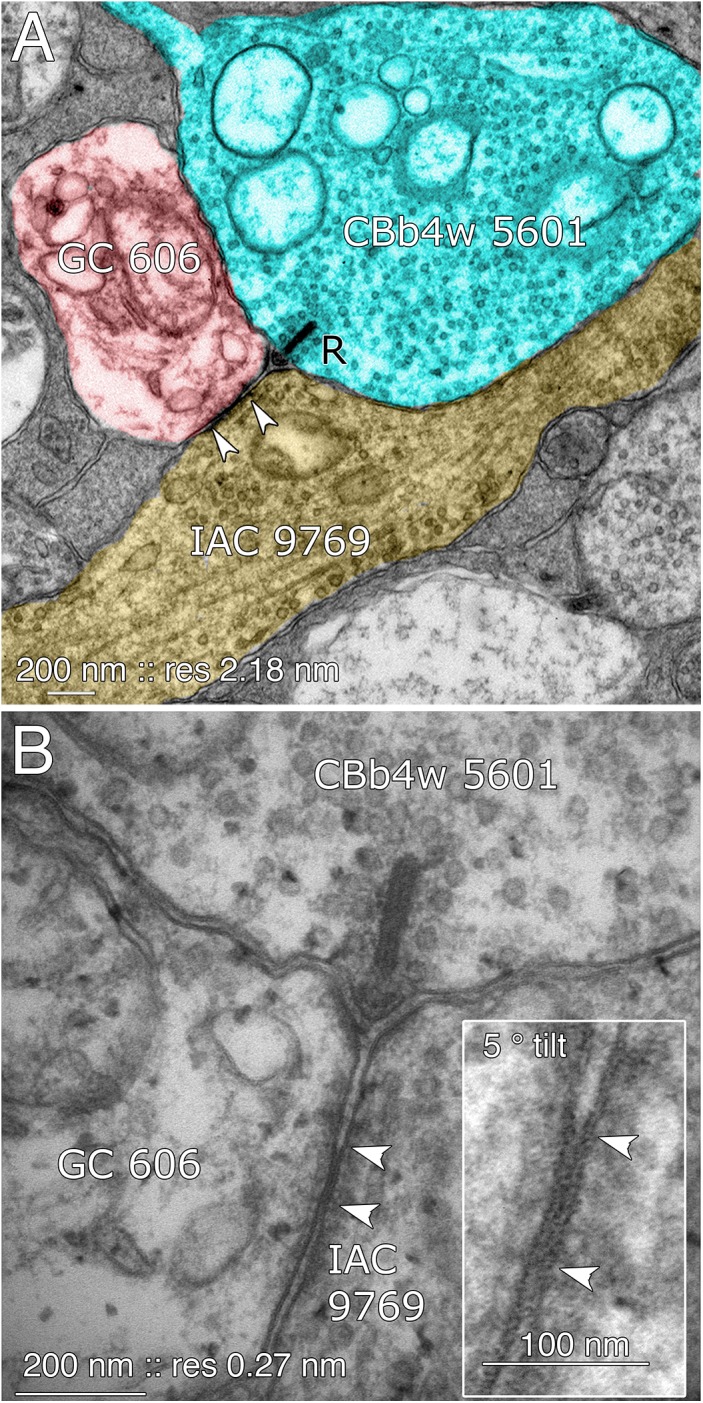
Coupling between GC 606 and IAC 9769. **(A)** Connectome RC1 image of CBb4w 5601 (cyan) providing dyadic synaptic ribbon (R) input to GC 606 (red) and IAC 9769 (yellow). A large gap junction between GC 606 and IAC 9769 is visible as a unique dense line over the apposed membranes of the two cells (bracketed by arrowheads). This is the basic identification schema for identifying gap junctions in the RC1 volume at its native 2.18 nm/pixel. Note that the gap junction can be “zoomed” to subpixel image levels in practice for annotating it (Anderson et al., 2011a). **(B)** TEM reimaging of the same gap junction and ribbon complex visualized at high resolution (0.27 nm/pixel) and goniometrically tilted 5° to optimize the multilaminar gap junction structure (inset). Reprinted by permission from (Marc et al., 2013).

**FIGURE 9:**
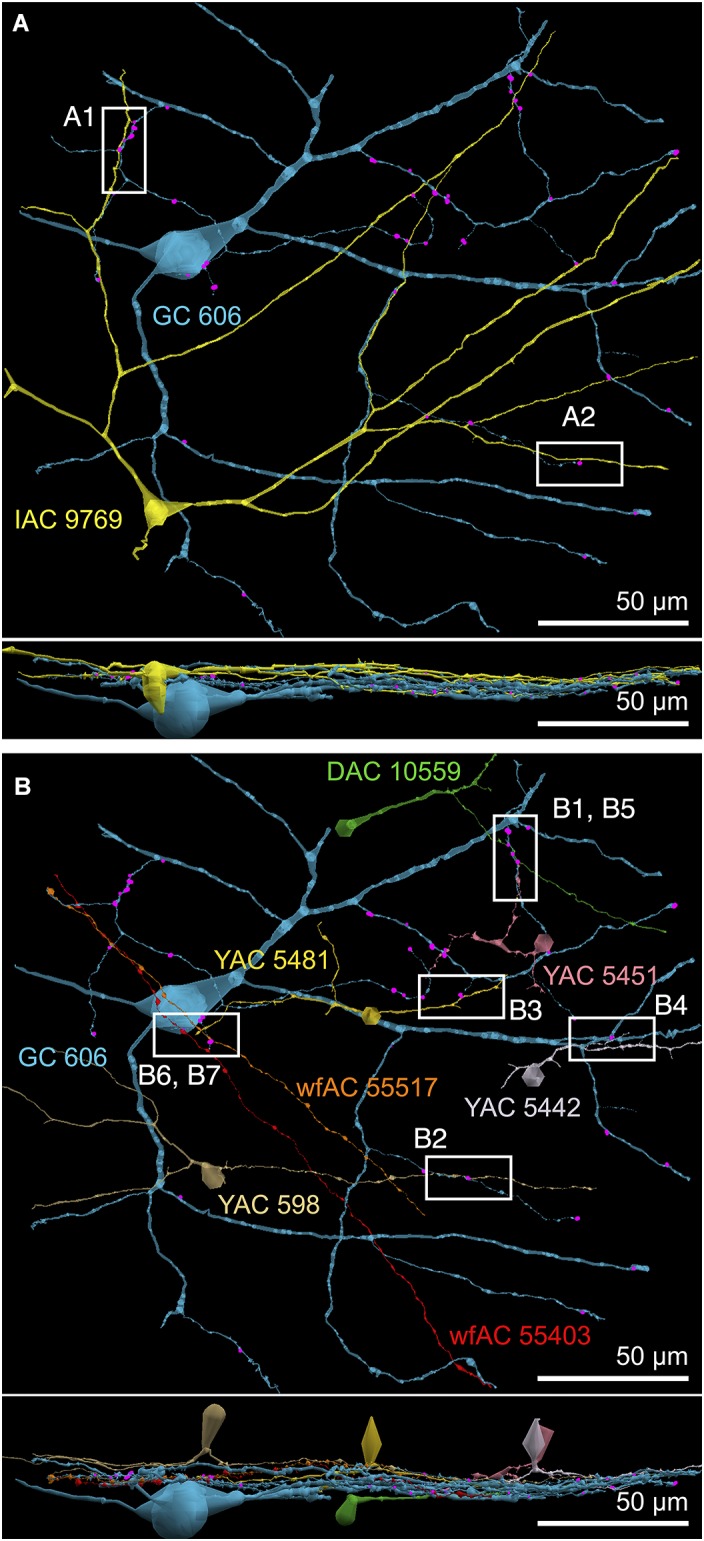
Selected sites of heterocellular coupling between inhibitory amacrine cells and GC 606. **(A)** Two loci of coupling (A1, A2) between IAC 9769 and GC 606 viewed as a horizontal field. Lower image, vertical overlay. **(B)** Seven loci of coupling between displaced amacrine cell (DAC) 10559; γ+ amacrine cells with somas in the RC1 volume (YACs) 5481, 5442, 5481, and 598; and wide-field γ+ amacrine cell (wfAC) processes 55403 and 55517 arising from somas outside the volume. Horizontal and vertical overlays. High resolution analyses of these loci are shown in Figure 10.

**FIGURE 10:**
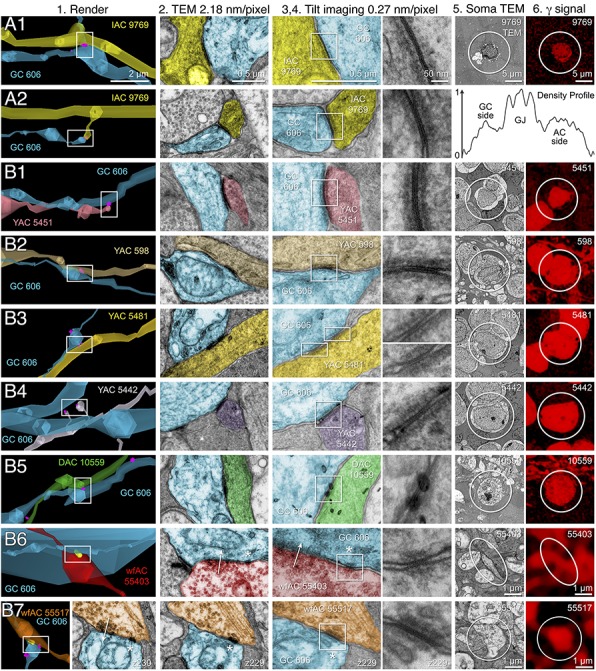
High-resolution analysis of coupling loci in Figure 9 imaged as: Column 1, VikingPlot renders; Column 2, RC1 native TEM at 2.18 nm/pixel; Columns 3 and 4, Goniometric reimaging at 0.27 nm/pixel; Column 5, soma or major process TEM; Column 6, GABA (γ) signal from the nearest intercalated CMP channel (Anderson et al., 2011b). **(A1)** Gap junction between GC 606 (cyan) and IAC 9769 (yellow). **(A2)** Gap junction between GC 606 (cyan) and IAC 9769 (yellow). Inset in columns 5,6 show a normalized plot of membrane density spanning the entire junction starting from the paramembranal density in GC 606, crossing the trilaminar zone and ending in IAC 9769 (ImageJ). **(B1)** Gap junction between GC 606 (cyan) and γ+ amacrine cell YAC 5451 (pink). **(B2)** Gap junction between GC 606 (cyan) and γ+ amacrine cell YAC 598 (tan). **(B3)** Gap junction between GC 606 (cyan) and γ+ amacrine cell YAC 5481 (yellow). **(B4)** Gap junction between GC 606 (cyan) and γ+ amacrine cell YAC 5542 (lavendar). **(B5)** Gap junction between GC 606 (cyan) and displaced γ+ amacrine cell DAC 5451 (green). Note that the lamination of the gap junction can be visualized through the inadvertent stain debris in column 4. **(B6)** Gap junction and adjacent conventional synapse between GC 606 (cyan) and wf γ+ amacrine cell wfAC 55403 (red). **(B7)** Gap junction and adjacent conventional synapse between GC 606 (cyan) and wf γ+ amacrine cell wfAC 55517 (orange).

The key feature that distinguishes GC 606 is its extensive and obvious coupling with amacrine cells and IACs (Figure 8). The morphology of retinal gap junctions is characteristic of vertebrate CNS, yielding multilaminar profiles at 0.27 nm/pixel resolution with spacing identical to those reported by Marc et al. (1988) using ≈0.1 nm resolution on film. In parsing the GC arbor contained within RC1, it is clear that GC 606 makes abundant small gap junctions with amacrine cell-like processes (Table 2). Of the 228 gap junctions, 61 have been successfully traced to specific source amacrine cells or IACs. The mean gap junction diameters for the entire cohort (181 ± 56 nm) are not significantly different from those of the identified amacrine cell subset (paired homoscedastic *T*-test, *p* = 0.43, dof = 284). The diameter range is 72–357 nm, and many gap junctions are thus sub-optical. All but two of the GC gap junctions in the entire volume RC1 are associated with amacrine cell processes and every GC::AC gap junction that is associated with a complete soma or traverses a reference slice arises from a GABAergic amacrine cell. Figure 9 illustrates the arbor of GC 606 and its overlap with coupled partner IAC 9769 (Figure 9A) and an additional set of seven coupled amacrine cells (Figure 9B). Key locations where representative gap junctions are formed are marked as A1, A2, B1, B2, etc. and displayed in Figure 10.

While the arbor of IAC 9769 coarsely intertwines with GC 606 at several loci, *fasciculation doesn’t correlate with the occurrence of gap junctions*, which typically appear at brief crossing points where the processes align for less than a few μm and even then gap junctions do not occur along the apparent alignment (Figure 9A). From a TEM perspective, gap junctions occur at loci where gaps in suboptical glial processes expose the target, similar to axonal ribbons in BCs (Lauritzen et al., 2012). IACs are not the only γ+ neurons that couple with GC 606. A set of conventional amacrine cells driven by CBb BCs are also coupled to GC 606 (Figure 9B). While their reconstructed fields are too limited to classify them all, they mostly appear to be wide-field (wf) γ+ amacrine cells, and there may be two or three classes that couple to GC 606. Representative validated gap junctions from IAC 9769 and the other γ+ amacrine cells are shown in Figure 10. At high resolution, it is clear that most gap junctions are not fasciculations but rather crossings (Figure 10 Column 1). The resolution of RC1 (2.18 nm/pixel) is sufficient to reliably detect gap junctions and measure their areas (Figure 10 Column 2) but is not adequate for complete validation as occasional adherens junctions can mimic gap junctions in oblique view (Marc et al., 2014b). High resolution (0.27 nm/pixel) reimaging with goniometric tilt allows validation of gap junctions by visualizing their characteristic multilaminar density profiles (Figure 10 Columns 3, 4 and inset). Finally, all of these coupled neurons are GABAergic (Figure 10 Columns 5, 6). Of course it is not possible to reimage every structure in every grid, but of the more than 2000 partner-identified gap junctions tagged in RC1 at 2.18 nm/pixel resolution, ≈20 have proven to be mistaken adherens junctions (<1% error) through tilt series recapture.

The real advantage of TEM connectomics database analysis is that we can take additional network hops and ask what the roles of the coupled interneurons might be. Every cell that is coupled to GC 606 is exported as a ^∗^.tlp format and its embedding network displayed in the Tulip framework^9^. All the amacrine cells coupled to GC 606 receive excitatory drive from CBb3, CBb3n, CBb4, and/or CBb4w ON cone BCs but not from the CBb5 and CBb6 cells that drive ON starburst amacrine cells and sustained ON and transient ON-OFF DS GCs. Thus, all are ON γ+ amacrine cells with matched input cone BC drive to that of GC 606.

**FIGURE 11:**
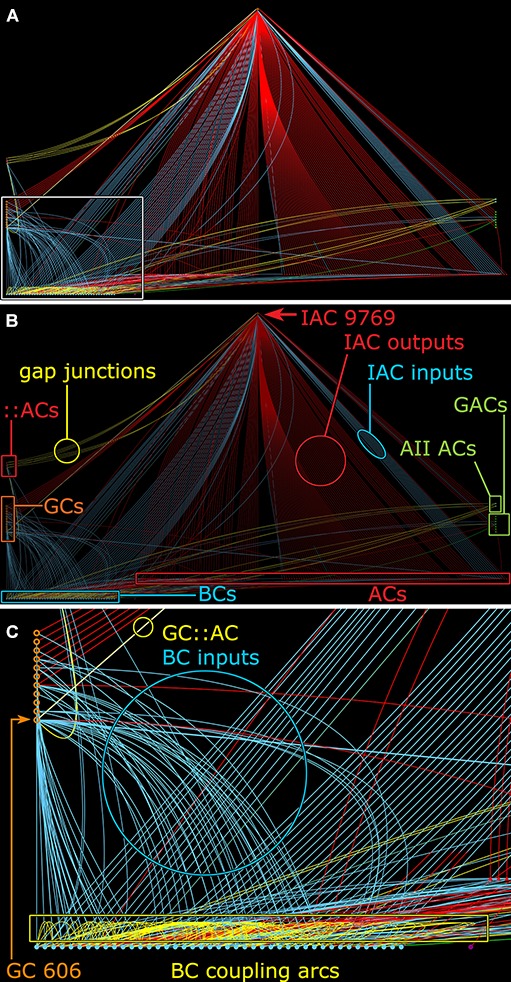
Graph of synaptic and gap junctional connectivity from IAC 9769 to amacrine, bipolar and ganglion cells. Tulip query showing bundled (i.e., multiple synaptic paths between loci are represented as single lines) dendrograms. Each line in the dendrogram represents a connection from IAC 9769 to another cell. **(A)** Annotation-free view with **(C)** inset marked (box). **(B)** Key: Red symbols, IAC, γ+ ACs and unidentified ACs; green symbols, A_II_ and GACs (glycinergic amacrine cells); blue symbols, bipolar cells; orange symbols, ganglion cells; small red symbols at left, IAC coupled ::ACs (coupled amacrine cells); red lines and arcs, synaptic outflow from IAC 9769; blue lines and arcs, synaptic input to IAC 9769 and instances of bipolar cell; yellow lines and arcs, gap junctions. **(C)** Enlargement of inset in **(A)**. GC 606 is strongly coupled to IAC 9769 (circled yellow edge, GC::AC) and other γ+ amacrine cells (yellow arc). Massive coupling networks exist among ON cone bipolar cells (yellow box).

Many ON γ+ amacrine cells are predominantly feedback amacrine cells that target ON cone BCs. Consistent with this, the density of feedback synapses in the ON cone BC networks in the entire connectome RC1 appears ≈3:1 higher than feedforward synapses: 2359 feedback synapses from amacrine cells onto BCs, 336 feedforward synapses by amacrine cells onto GCs and 564 feedforward synapses by amacrine cells onto other amacrine cells. This lumped analysis masks the exceptional specificity of various well-known cells. For example, rod BC-driven A_I_ amacrine cells are exclusively feedback amacrine cells, and the cohort of A_I_ amacrine cells in RC1 make 837 feedback synapses onto BCs and 0 feedforward synapses to either GCs or other amacrine cells. In contrast, the specific cohort of ON γ+ amacrine cells coupled to GC 606 also shows direct feedforward to GCs other than GC 606 with morphologies and circuities inconsistent with the tON DS GC classification. For example, IAC 9769 has a strongly reversed bias (>10:1 feedforward:feedback), targeting 38 amacrine cells and 13 GCs but only 3 cone BCs (Figure 11).

**FIGURE 12:**
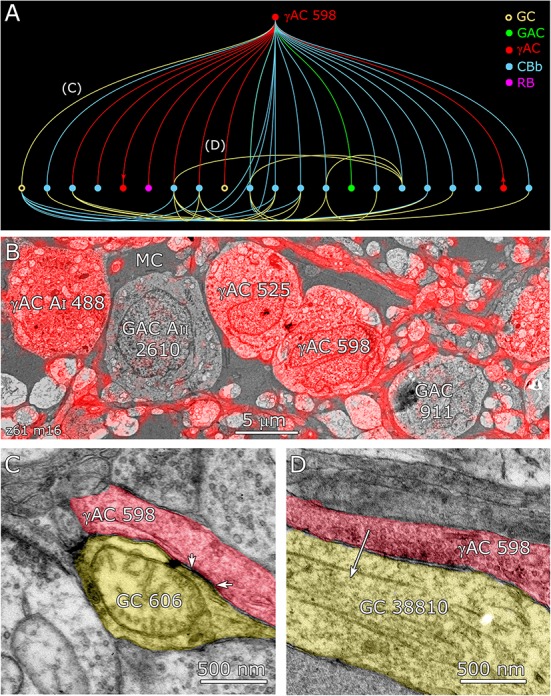
Coupling flow from GC 606 through γ+ amacrine cell 598 to multiple targets. **(A)** Tulip query bundled dendrogram plot of all the sources and targets of amacrine cell 598: GC yellow circles, ganglion cells: GAC green dots, glycinergic amacrine cells; γAC red dots, GABAerigc amacrine cells; CBb cyan dots, ON cone bipolar cells; RB magenta dot, rod bipolar cell. Line color denotes the presynaptic source. Arrows denote presynaptic source in γAC to γAC paths. Each line represents a bundle of synaptic lines. **(B)** Validation of GABAergic identity for AC 598. **(C)** A gap junction between AC 598 (red) and GC 606 (yellow) delimited by arrowheads. **(D)** Synapse from AC 598 (red) to GC 38810 (arrow).

Feedforward does not send inputs recursively into the upstream network like feedback does, allowing for strong channel isolation even if the interneuron is involved in feedback. An excellent example is ON γ+ AC 598 (Figure 9B) which engages in both feedforward and feedback, transferring sign-conserving coupled signals from GC 606 via sign-inverting GABA synapses to another ON GC (Figure 12).

The coupled set of identified γ+ IACs/ACs and additional unclassified ACs form over 200 gap junctions with GC 606 *in the RC1 volume*, implying that the complete cell forms over 1000 gap junctions, thereby comprising a massive coupling path between the inhibitory and excitatory networks of the retina. Cross-class inhibitory feedforward driven by coupling to GC 606 converges on pure ON GCs (ID 7594, 15796) and ON-OFF GCs (ID 5107, 6857). ON GC 7594 is also γ+ (Figure 3), albeit at lower levels than GC 606, but none of the GC 606-coupled amacrine cells appear to couple with GC 7594. ON-OFF GC 5107 is uncoupled and γ-, while ON GC 15796 is very weakly γ+ and GC 6857 is strongly γ+. Thus, this feedforward inhibition does not appear to discriminate GC classes. We can mathematically summarize this chain as: GC1 :: AC > _i_GC2 (where class 1 ≠ class 2, i.e., they are *disjoint* sets). Other GCs receiving feedforward input in connectome RC1 are too incomplete to classify as they arise from outside the volume and it is impossible to connect branches to exclude mixed polarity inputs. Those with pure OFF inputs (OFF GCs) remain a possibility. For example, GC 606-coupled γ+ AC 5451 is pre-synaptic to GC 28950, an unbranched process traversing the OFF layer with only OFF cone bipolar inputs. If we use the rough scaling for size obtained in Table 2, a target GC could receive at least 200 inhibitory synapses via a single amacrine or axonal cell, driven by a coupled GC of a different class. This must be a vast underestimate, since we cannot trace the majority of the coupled processes that arise from outside the volume.

Finally, we have found no proven homocellular gap junctions between GCs. This is consistent with findings in mouse retina that homocellular coupling is always in-class, never cross-class (Völgyi et al., 2009, 2013b; Pan et al., 2010). There are 2 candidate junctions out of many thousands of gap junctions in the RC1 connectome, but we cannot validate the processes as GCs. The lack of homocellular coupling in connectome RC1 does not mean it does not exist in rabbit, since the RC1 volume is too small to ensure discovery of coupling between the small overlap zones of GCs of the same class.

### GC 9787

**FIGURE 13:**
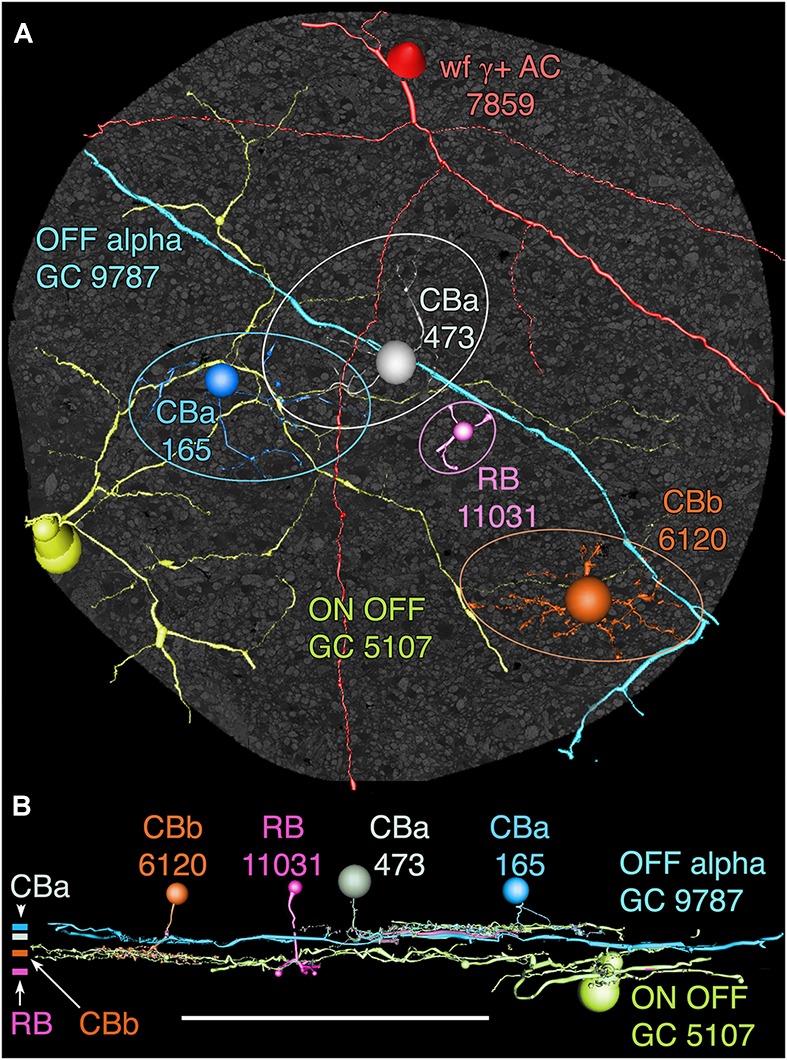
OFF alpha ganglion cell candidate dendrite GC 9787 crosses the connectome volume. **(A)** Horizontal view of OFF alpha GC 9787 (cyan) dendrite in comparison to ON–OFF GC 5107 arbor (yellow–green) and four reference bipolar cells. ON–OFF GC 5107 is driven by both ON cone bipolar cells (e.g., CBb3n 6120 tangerine) and OFF cone bipolar cells (CBa 165 blue) that bracket the OFF inner plexiform layer and are distal within the inner plexiform layer to the rod bipolar cell terminals (e.g., RB 11031, magenta). GC 9787is driven by a separate set of more proximal OFF cone bipolar cells (e.g., CBa 473 gray). Ellipses delimit bipolar cell axonal fields. **(B)** Vertical view displaying the separate strata for rod (RB), ON cone (CBb) and OFF cone (CBa) bipolar cells. CBa 473 that drives OFF alpha GC 9787 is proximal to the OFF CBa 165 and similar bipolar cells that drive GC 5107.

**FIGURE 14:**
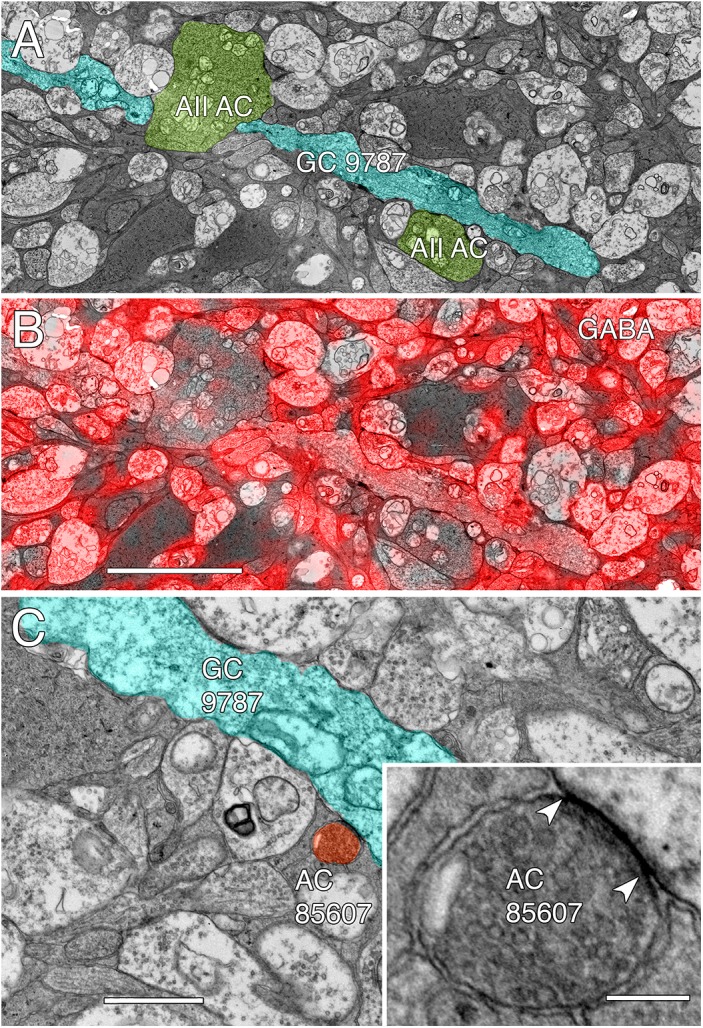
GABA-coupled signals in GC 9787. **(A)** Single TEM slice z184 containing a segment of GC 9787 (cyan) flanked by AII AC lobules (yellow–green). **(B)** The same TEM image from slice z184 overlayed with the neighboring intercalated GABA signal showing positive colocalization. Scale 5 μm. **(C)** Typical gap junction between GC 9787 (cyan) and an amacrine cell (AC 85607) at a crossing, non-fasciculated junction. Scale 1 μm. Inset. High resolution image of the gap junction, showing characteristic gap occlusion. Scale 100 nm.

Among the full cohort of GCs, OFF alpha GCs in the rabbit retina are characterized by a number of key features. In peripheral retina (rabbit volume RC1) they are among the largest of retinal GCs with very large, simple dendrites of 1–2 μm diameter, dendritic arbors of ≈0.5–0.9 mm, somas approaching 30 μm in diameter and extensive heterocellular coupling to amacrine cells (Xin and Bloomfield, 1997; Marc and Jones, 2002; Peichl et al., 2004). The somas can protrude deeply into the inner plexiform layer and insert large dendrites into the OFF layer of the inner plexiform layer. Additionally, they receive extensive input from both OFF (CBa) cone BCs and A_II_ ACs (Kolb and Famiglietti, 1975; Marc et al., 2014a). None of the GCs with somas positioned in the RC1 volume fit this profile. Due to the sparse but uniform coverage of OFF alpha GC dendrites, we presumed that the largest crossing dendrite of the OFF layer in volume RC1 was the most probable candidate to be from an OFF alpha GC: GC 9787 (Figure 13). GC 9787 is a large, 1.5 μm diameter process traversing the proximal half of the OFF stratum, while reference ON-OFF GCs, e.g., GC 5107 have their dendrites and input OFF BC terminals in the most distal portion of the inner plexiform layer. Because the dendrite exhibits a single branch along the entire stretch of its crossing volume RC1, it likely represents a cell with a much larger field than nearby bistratified diving GC (Lauritzen et al., 2012) and even tON DS GCs, and clearly excludes classification as a classical X-type sustained GCs and a range of W-type cells, even those that are coupled. Beyond size, four features suggest that this single large dendrite crossing the volume arises from an OFF alpha GC. First, it collects inputs only from a subset of OFF cone BCs (mostly CBa2), especially at multi-ribbon, large PSD sites (Supplementary Figure S1), but not CBa1 and CBa1w BCs and axonal ribbons of ON cone BCs in the OFF layer. In contrast, dendrites of bistratified diving GCs traverse the OFF layer collecting OFF-layer axonal ribbon inputs from ON cone BCs but never inputs from the resident OFF cone BCs (Lauritzen et al., 2012). Interestingly, GC 9787 appears to form large PSDs (up to 600 nm diameter) only at pre-synaptic ribbon sites (Supplementary Figure S1) and never at conventional (ribbonless) BC pre-synapse sites, which are, in fact, quite common in the OFF layer and formed by the same BCs onto different targets (Anderson et al., 2011b; Marc et al., 2013). For example, bistratified ON-OFF GC 8575 collects two *conventional ribbonless* OFF BC synapses for every OFF ribbon it contacts. Second, GC 9787 receives conventional inhibitory chemical synapses from every AII AC it encounters, six cells in all across the volume (Supplementary Figure S2). Third, the process traverses GABA-labeled slice z184 in the RC1 volume multiple times and is clearly γ+ (Figure 14), making it an excellent candidate for heterocellular coupling with γ+ amacrine cells. Finally, it forms distinct gap junctions with amacrine cells (Figure 14C).

Except for the southeast margin of the volume, GC 9787 is a smooth, unbranched dendrite very unlike the topology of GC 606 and represents only ≈0.3 mm of length. The entire passage of GC 9787 through the OFF layer collects 13 gap junctions with an area of 9142 nm^2^/μm of dendrite length, which is ≈46% of the gap junction density of GC 606. The frequency and range of size of gap junctions formed by GC 9787 (≈0.37 ± 0.19 μm^2^) tend to be on the larger size of gap junctions in RC1, but this sampling is not significantly different from those gap junctions formed by GC 606 (≈0.28 ± 0.18 μm^2^) as assayed by either parametric (unpaired, heteroscedastic *t*-test; *F*-test) or non-parametric (Kolmogorov–Smirnov) measures. However, as any power calculation is defined by the smallest sampling group (gap junctions in GC 9787), the calculated power only reaches ≈0.3 with α = 0.05, and the false negative rate β is very high at 0.7. So, it is very possible that the gap junction sizes between GCs *are* significantly different, especially since the GC 606 statistics are stable (due to the very large sample) and its coefficient of variation is stable to less than 25% of a decimated sample set.

The cohort of coupled amacrine cells exclusively receive input from OFF cone BCs. Whether the class distribution of these excitatory inputs matches that of GC 9787 will have to wait for more detailed classification of the OFF cone BC cohort, but like GC 9787, these amacrine cells exclusively receive this input via ribbon-containing pre-synaptic sites. The set of GC 9787-coupled amacrine cells includes two γ+ amacrine cells: a large, γ+ monostratified OFF AC (YAC 7859, Figure 13A) and a long, unbranched amacrine cell process whose soma lies outside the RC1 volume. While we cannot verify that every coupled process is γ+, there is no evidence that any glycinergic amacrine cell in RC1 is coupled to either GC 9787 or GC 606. While we previously identified a single candidate glycine- and GABA-coupled GC class in the rabbit retina (Marc and Jones, 2002), we have not yet encountered a valid instance of glycinergic amacrine cell coupling to GCs in RC1.

Similar to GC 606, there is feedforward signal flow from GC 9787 via coupled OFF γ+ amacrine cells to both GC 9787 itself and other short fragments of non-alpha GC dendrites in the OFF layer. Some non-alpha dendrites are themselves γ+. At least one of these does not form gap junctions with these same amacrine cells (ID 43716), implying that they may be coupled to different sets of amacrine cells, as is the case with GC 606. However, with both the GC and amacrine cell extending processes beyond the volume boundaries of RC1, it is possible that such coupling occurs elsewhere in their arbors. Two GC processes do couple with these same amacrine cells. Both (ID 28950, 5150) are also large-caliber single- or un-branched processes and receive frequent input from AII amacrine cell lobules, not inconsistent with OFF alpha dendrites. The high overlapping coverage of adjacent OFF alpha dendritic arbors (Völgyi et al., 2005) therefore makes it impossible to rule out that these are OFF alpha dendrites from the same or other OFF alpha GCs. While a complete tabulation of the connectivity of coupled OFF amacrine cells would require over a year’s worth of dedicated annotation and classification time, Tulip queries reveal that some partners such as wf γ+ AC 7859 appear to be biased toward feedforward contacts, similar to IAC 9769 in the ON system, and support cross-class inhibitory feedforward to other GC classes.

## Discussion

### GABA Signatures

GABA content is a useful signature for predicting coupling in the GC layer. There have been no known GABA transporters described on any GCs, much less GCs in the adult rabbit retina (Hu et al., 1999). Unlike coupled GCs, uncoupled cells have no GABA signal but all GCs have mathematically inseparable glutamate signatures (Marc et al., 1990, 1995; Marc and Jones, 2002), regardless of GABA content (Figure 1E). In support of this observation, we have found no gap junctions made by any γ- GCs in RC1. GCs display GABA levels ranging from undetectable to close to *bona fide* amacrine cell levels (Marc and Jones, 2002), with the majority centered around 300–600 μM, 10-fold lower than typical starburst amacrine cell levels (Figure 1F). Given that specific GCs show extensive heterocellular coupling with markers such as Neurobiotin (MW 322), it is not surprising that a molecule several times smaller, such as GABA (MW 103), is highly mobile through gap junctions, similar to quantitative measures of glycine coupling into ON cone BCs from glycinergic AII amacrine cells (Marc et al., 2014a). Glycine is present in appreciable amounts in BCs despite the lack of a synthesizing enzyme and transporter, explicitly due to AII amacrine cell coupling. Indeed, for other work, we use glycine as an index on ON cone BCs revealing their coupling to AII amacrine cells (Marc et al., 2014a). Importantly, GCs known to show heterocellular coupling such as rabbit OFF alpha GCs (Xin and Bloomfield, 1997) and tON DS GCs (Hoshi et al., 2011) uniformly show intermediate GABA levels (Marc and Jones, 2002). Importantly, Ackert et al. (2006, 2009) and Hoshi et al. (2011) also demonstrated that an axonal cell (axon-bearing “amacrine” cell) virtually identical in dendritic morphology to our IAC 9769 was both coupled to tON DS cells and γ+ by immunocytochemistry, and that other amacrine cells with differing arbor patterns were also coupled. In contrast, GCs that we know are definitively not dye-coupled, such as primate midget GCs (Dacey and Brace, 1992), never show GABA coupling (Kalloniatis et al., 1996).

This sets the framework for using glutamate and GABA as markers of GC coupling in other species (Figure 2), since antibodies targeting small molecules have no species bias. In surveying our library of all vertebrate classes and many vertebrate orders (Supplementary Table S2), we find that apparent heterocellular coupling between GCs and amacrine cells is widespread with only one group failing to show evidence of coupling: *Trachemys scripta elegans* (formerly Genus *Pseudemys*), Order Testudines, Class Reptilia. As all vertebrate classes show evidence of heterocellular GC::AC coupling, this argues for such signaling as a feature of primitive retina and perhaps even of its predecessor diencephalic primordia. Indeed, extensive coupling and regions of high cell firing synchronicity is a hallmark of early mammalian brain differentiation (Niculescu and Lohmann, 2014). In sum, these considerations argue for heterocellular GC::AC coupling as a retinal *plesiomorphy* (a basal feature of ancient retinas), not a synapomorphy (specialization of a clade) among select classes, and that heterocellular coupling is foundational for the retina as argued by Völgyi et al. (2013a).

### Coupling and Feedforward

But what is the functional network role of heterocellular coupling? A fundamental clue arose when certain retinal GCs and downstream neurons in brain were found to show synchronized spiking across cells (Alonso et al., 1996; Hu and Bloomfield, 2003) and that the mostly narrowly correlated retinal firing persisted after global pharmacologic synaptic suppression, leading to the argument that it was mediated by coupling (Brivanlou et al., 1998). Subsequent analyses have refined these concepts to show that correlated spiking appears to occur within sets of the *same* GC class, including the OFF alpha GC class, and that one essential pathway is heterocellular coupling (Völgyi et al., 2013b; Roy et al., 2017). Our findings are consistent with this view: (1) homocellular cross-class GC coupling is non-existent in connectome RC1, (2) heterocellular coupling between GCs and multiple classes of amacrine cells is abundant and robust, and (3) in instances where multiple GC processes couple to the same amacrine cell, the ganglion processes are not obviously of different classes. No evidence emerged to show that any amacrine cells in the coupling networks for one tON DS GC and one OFF alpha GC are shared: they seem completely separate. However, we do find sparse instances of coupling among the γ+ ACs coupled to GC 606, but these short fragments are impossible to classify and may reflect homocellular coupling between amacrine cells of the same class, which has been supported, for example, for the IACs due to their robust tracer coupling (Wright and Vaney, 2004) and Neurobiotin visualization using photochromic 2-stage intensification as described in Vaney (1992).

Interstitial amacrine cells and ON γ+ amacrine cells coupled to tON DS GCs share the same profile of excitation: a bias for class CBb4w ON cone BCs and against classes CBb5 and CBb6 cells that drive starburst amacrine cells (Figure 7). While our analysis of OFF cone BC populations is not yet as refined as for ON cone BCs, the excitatory drive to amacrine cells coupled to OFF alpha GCs shares similar biases: toward OFF Cba2 BCs and away from CBa1 BCs. Considering the high diversity of vertebrate amacrine cell classes (Wagner and Wagner, 1988; MacNeil et al., 1999), every instance of GC::AC coupling could easily involve unique sets of amacrine cells for each coupled GC class, though such class separation may not be essential.

**FIGURE 15:**
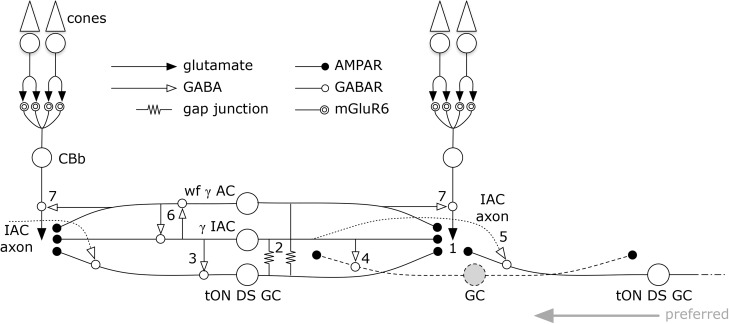
Signal flow through the tON DS GC :: γ+ AC network. Key in inset. (1) ON cone bipolar cell signals are collected by all cell classes at AMPARs or AMPARs + NMDARs. GC :: AC gap junctions connect networks of (2) γ+ IACs and wide-field (wf) γ+ ACs. IACs are predominantly feedforward, driving sets of ganglion cells including (3) the coupled tON DS GC, (4) local dendrites from GCs outside this coupled set, and (5) distant instances of tON DS GCs in the far surround via their axons. IACs also engage in (6) nested feedback with wf γ+ ACs, which are themselves mixed feedforward (not shown) and (7) feedback inhibitory neurons. This model may also support a directional bias for tON DS GCs with the preferred direction arising from the regions driven by the axonal field of distant IACs.

But amacrine cells are not simply conduits for coupling. Every amacrine cell class that we know well is either GABAergic or glycinergic. Indeed, every amacrine or axonal cell involved with heterocellular GC::AC coupling whose signature can be retrieved is GABAergic. And connectomics can resolve the targets of these coupled amacrine and axonal cells. Importantly, both ON and OFF instances of GC::AC coupling demonstrate feedforward synapses directly from coupled ACs to *different* classes of GCs: cross-class inhibition. As schematized in Figure 15, heterocellular coupling allows an active GC to directly inhibit its neighbors: GC1::ON AC > _i_ GC2; where >_i_ denotes sign-inverting signaling, :: denotes coupling and classes GC1 and GC2 are disjoint. The essential feature is that inhibitory postsynaptic currents (IPSCs) should be generated in a halo of different GC classes closely synchronized with the spikes of a source GC. If these IPSCs were strong enough to suppress some incidentally coincident spikes in target GCs, this could create an improved signal-to-noise ratio (SNR) at the CNS downstream targets of the source GC compared to a parallel channel (Figure 15). Certain GCs (e.g., ON–OFF DS GCs) show Na-dependent dendritic spiking (Oesch et al., 2005; Schachter et al., 2010), potent spike veto by inhibitory processes (Sivyer and Williams, 2013) and postsynaptic current integration (Brombas et al., 2017). This argues that dendritic inhibition in GCs can be strong enough to suppress dendritic spiking. The bleed-through of excitation from the tON DS GC into a set of GABAergic neurons that target different GCs means that such heterocellular coupling likely has the ability to suppress activity in nearby disjoint populations.

While the potential for precise timing of both synchronized spikes and feedforward inhibition is clear, it is also certain that many wf γ+ amacrine cells (e.g., Figure 12) provide feedback to cone BCs of matched polarity: ON AC > _i_ ON CBb and OFF AC > _i_ OFF CBa. This provides a much broader fan-out of targets for the GC::AC inhibitory couple, amplified explicitly by the positive gain of BC ribbon synapses (e.g., Lauritzen et al., 2016).

Heterocellular coupling between spiking projection neurons and local inhibitory neurons may be more widespread than appreciated. Like retina, olfactory bulb generates synchronized oscillatory excitation/inhibition interactions that are enhanced by Cx36-mediated coupling (Pouille et al., 2017) though the initial mechanism was modeled as homocellular coupling of mitral cell (MC) pools. However, detailed analysis of inhibitory intraglomerular networks provide strong evidence for heterocellular coupling between mitral cells and specific short axon GABAergic cells in olfactory glomeruli and that the coupling, at least, is part of the mechanism that truncates events to permit more precise excitation/inhibition coordination in mitral cells (Liu et al., 2016). This may be a common mechanism in many “transient” neurons as it is consistent with feedforward onto the coupled source in both instances of GCs, albeit with completely different inhibitory networks. Similarly, physiological evidence supports an analogous network for timing control in olfactory bulb. While little is known of the cell class distinctions among neighboring mitral cells in olfactory bulb, there is strong evidence for multiple projection classes, intrabulb short-range excitatory projections, and inhibitory classes including different classes of GABAergic short axon cells (Nagayama et al., 2014). We would predict that specific classes of short axon neurons (SANs) make heterocellular gap junctions with specific mitral cells and inhibit neighboring mitral cells where MC1 and MC2 are disjoint sets: M1 :: SAN > _i_ M2.

Finally, coupling networks involving inhibitory neurons can take on very complex frequency-dependent operations, such as Golgi neurons in cerebellum (Vervaeke et al., 2010, 2012; Pereda et al., 2013). In a similar fashion, it is plausible that the IAC might not have effective dendritic spiking and are more passive cables, like cerebellar Golgi interneurons, but the high density of GC::AC coupling acts as an excitation repeater. Further, such networks could either enhance synchrony or desynchronize in different excitation modes (Vervaeke et al., 2010).

### Arbor Size and Resolution

There is a major caveat arising from the conflicting demands of connectomics coverage and resolution. Once captured, one can downsample but never upsample, one can mine an area but never expand. Dedicating more bits to one mode steals from the other. Unlike small-field BCs and glycinergic amacrine cells, GCs and GABAergic neurons can have arbors much larger than a connectome. Ackert et al. (2009) showed the axonal cells coupled to ON DS GCs had fine extensions of their terminal dendrites that ascended into the OFF layer and co-stratified with the OFF ChAT+ starburst amacrine cells. As IAC 9769 has long straight dendrites that exit the full volume perimeter, such unusual morphologic features cannot be excluded. Thus, there appears at least two crossover paths between the IAC and the OFF layer. In addition to the apparent cholinergic layer ramification, it is clear (via Tulip queries) that crossover glycinergic amacrine cells driven by OFF cone BCs also synapse on IAC 9769. The data of Ackert et al. (2009) do not reveal a glycinergic path, though it clearly exists. Their data support the OFF starburst amacrine cell path: OFF CBa > OFF SAC > IAC :: ON DS GC; where > denotes sign-conserving synapses and :: denotes coupling. It is remarkable that, despite abundant opportunity (Figure 4), the ON γ+ IAC completely rejects interaction with the ON starburst amacrine cell arbors in favor of the OFF arbors.

### Direction of Motion

The coupled tON DS GC is a largely separate stream of directional signaling with little apparent engagement with the ON-OFF DS cohort (Ackert et al., 2006; Hoshi et al., 2011), but does contribute to the classic three-lobed orientation distribution of ON DS GCs reported by Oyster and Barlow (1967). Consistent with this model, we find not only complete synaptic separation of tON DS GCs and the starburst amacrine cell network, but also nearly total separation of each other’s BC input profile. Nevertheless, like other DS cells, directional signaling by tON DS GCs is dependent on GABAergic inhibition and is suppressed after global GABA blockade (Ackert et al., 2006, 2009). Directional selectivity in visual cortex has been thought to be driven by asymmetries in excitation, although differing spatial distributions of excitation and inhibition clearly play a role (Li et al., 2017). IACs, as axonal cells, offer a built-in simple asymmetry that could be maximized for low velocity directional motion if: (1) their axons behave as classical axons and arborize into predominantly presynaptic terminals; (2) the axons do not form gap junctions; and (3) the axons target tON DS GCs. We simply don’t have information on the latter in connectome RC1, but it is important to consider two quantitative points. First, a complete tON DS GC likely receives over 4,000 inhibitory synapses and the bulk of those will be GABAergic (15-fold more prevalent than glycinergic synapses onto GC 606). The fact that coupled amacrine cells make up a small fraction of that inhibitory input via feedforward simply means that the bulk of inhibition is driven by sets of wide field amacrine cells or IAC axons arising from outside the volume, displaced from the centroid of the GC 606 arbor. Importantly, prior work had shown these IACs (also known as axon-bearing amacrine cells) had long sparse axons (Ackert et al., 2006, 2009; Hoshi et al., 2011) but some of these are incomplete, as a more complex terminal arbor was demonstrated in Massey (2008). A more complete description of bona fide IACs in primate by Dacey (1989) describe each IAC as being surrounded by a sparse halo of axon terminals roughly 10x the diameter of the dendritic arbor and yielding perhaps over 100 fold greater coverage. Similarly, IAC 9769 in RC1 is identical to the PA1 polyaxonal cell in rabbit meticulously described by both Famiglietti (1992) and Wright and Vaney (2004). In any case, there is ample additional space in the tabulation of synapses to accommodate sparse inhibitory cells with very high coverage factors, in addition to IAC axons. Wright and Vaney (2004) also show that the net density (length) of axonal processes is ≈10× higher than dendritic density. If this translates to synaptic density and the axon has the same target preferences as the IAC dendrites, it is very likely that a large fraction of inhibition targeting tON DS GCs could arise from the IAC or PA1 polyaxonal cell. We have not shown that the non-IAC processes coupled to GC 606 correspond to the diffuse multistratified cell previously described (e.g., Hoshi et al., 2011), but presuming we are selecting for parts of their arbors, this cell may be even better suited than the IAC for mediating cross-class inhibition. The second point is that a gap junction on GC 606 is never more than a micron away from a BC ribbon input, so the shunting path for any dendritic spikes is very short. This lays the framework, via IAC axons or wf amacrine cells, to provide narrowly shaped and time-locked, delayed feedforward IPSCs to other tON DS GC instances in the coupled neighborhood.

## Conclusion

Physiological and tracer studies have firmly established heterocellular coupling as a norm in the mammalian retina. By combining small molecule markers and connectomics we provide some additional insights. First, heterocellular GC::AC coupling is likely a *plesiomorphy* and not a synapomorphy. Second, in the instances of GC::AC coupling we know well in the mammalian retina, one involving tON directionally selective GCs and the other engaging transient OFF alpha GCs, the coupled GABAergic amacrine and axonal cells clearly inhibit many neighboring cells, including feedforward inhibition onto neighboring GCs of different classes, outside the coupled set. Thus, an activated GC may inhibit neighboring GCs of different classes in a time-locked fashion, potentially erasing coincident dendritic spikes across GC classes. If we can now begin to tabulate and explore the detailed distributions of inhibition relative to the sites of coupling, we may uncover spatial asymmetries that convert to temporal delays necessary for encoding direction in this unique cohort of ganglion cells.

## Author Contributions

RM wrote, edited, created figures, annotated and analyzed content for this manuscript. CS annotated and analyzed the data, created figures, and edited the manuscript. RP annotated and analyzed the data and edited the manuscript. DE extensively annotated. JA created connectome volume builds, maintained and edited annotation and image data in the database. BJ directs the research lab and science, edited the manuscript, participated in data generation, annotation, analysis, read and approved the submitted version.

## Conflict of Interest Statement

RM is a principal of Signature Immunologics Inc., the source of some of the antibodies used for this research. RM is a principal of Signature Immunologics, the source of some of the antibodies used for this research. The remaining authors declare that the research was conducted in the absence of any commercial or financial relationships that could be construed as a potential conflict of interest.

## Footnotes

1 connectomes.utah.edu

2 graphviz.org

3 tulip.labri.fr

4 Blender.org

5 https://github.com/visdesignlab/TulipPaths

6 https://docs.python.org/2/library/re.html

7 www.analystsoft.com/en/

8 www.r-project.org

9 tulip.labri.fr

## Supplementary Material

FIGURE S1 Multi-ribbon OFF cone bipolar cell inputs to GC 9787. Five serial TEM sections (z168–z172) are coded for GC 9787 (cyan) and CBa 165 (green). Over a span of 280–300 nm, at least 4 bipolar cell synaptic ribbons (R) dock presynaptically across from a single large postsynaptic density (bracketed by arrowheads). Arrows denote direction of synaptic flow (pre → post). Scale 1000 nm.

FIGURE S2 GC 9787 dendrites (cyan) collect multiple synaptic inputs from glycinergic AII amacrine cell distal lobular appendages (green) across the volume. **(A)** Conventional synapses from GAC AII 7113 onto both GC 9787 and CBa 35696 (tan) which is presynaptic to GC 9787 at two other sites. **(B)** Convergent signaling from γ+ amacrine cells (pink γ+ AC), GAC AII 284, and a CBa bipolar cell (tan) onto GC 9787. GAC AII 7157 makes synapses onto 9787 in another section (not shown) but is also presynaptic to the CBa bipolar cell. **(C)** Single synapse from lobule GAC AII 8032 onto GC 9787. **(D)** Classical multiple presynaptic densities associated with a single GAC AII synapse. Scales 1000 nm.

TABLE S1 Examples of retinal cell classes, intermediate groups and superclasses.

TABLE S2 GABA immunocytochemistry species list.

TABLE S3 Log10 relative ligand required to block tissue binding.
