# Supplementary figures and images for: Heterocellular Coupling Between Amacrine Cells and Ganglion Cells
Source: Front Neural Circuits. 2018 Nov 14;12:90. doi: 10.3389/fncir.2018.00090 (PMC6247779; doi:10.3389/fncir.2018.00090)

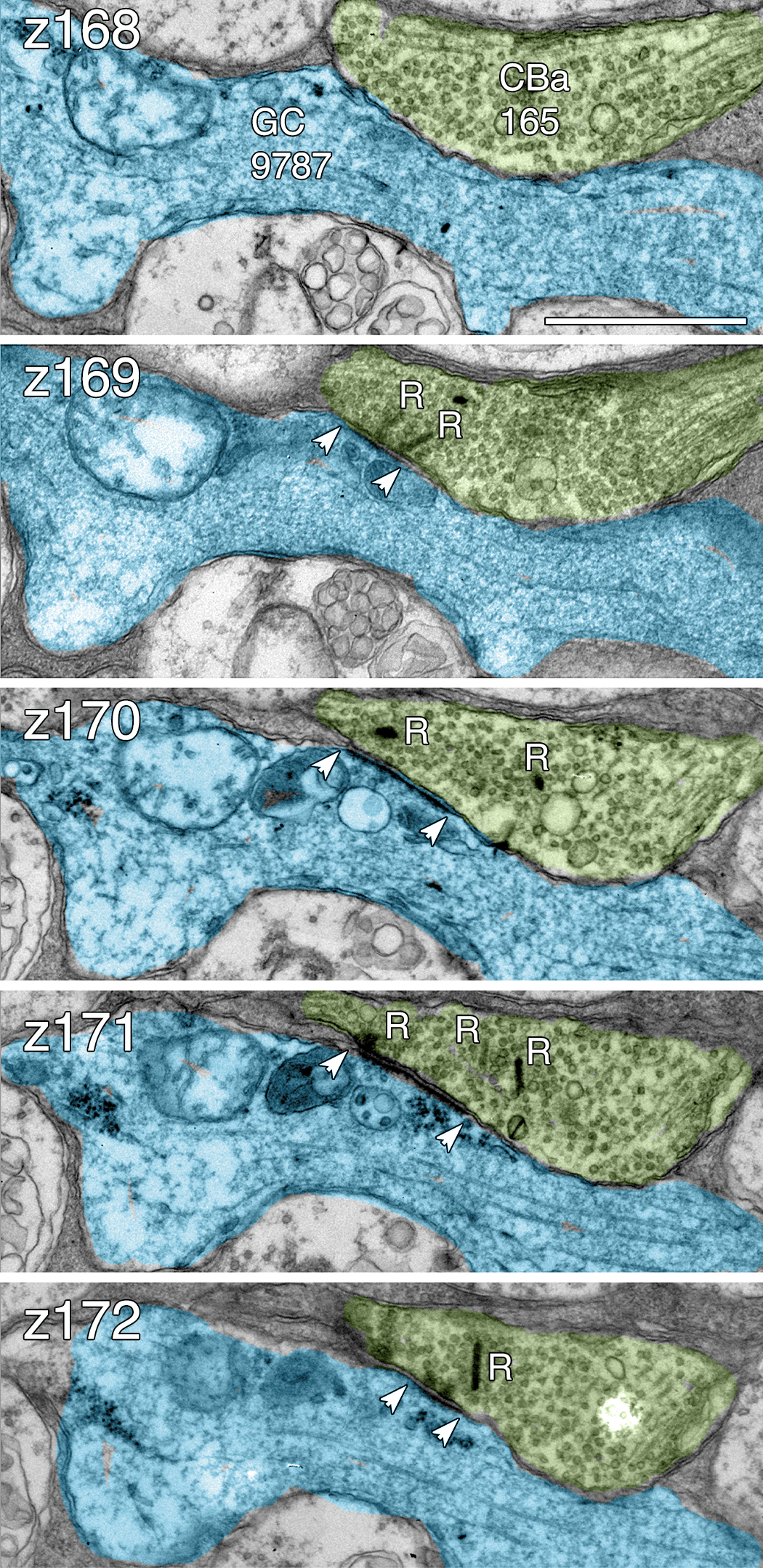

Supplement: FIGURE S1 — Multi-ribbon OFF cone bipolar cell inputs to GC 9787. Five serial TEM sections (z168–z172) are coded for GC 9787 (cyan) and CBa 165 (green). Over a span of 280–300 nm, at least 4 bipolar cell synaptic ribbons (R) dock presynaptically across from a single large postsynaptic density (bracketed by arrowheads). Arrows denote direction of synaptic flow (pre → post). Scale 1000 nm. [file Image_1.tif]

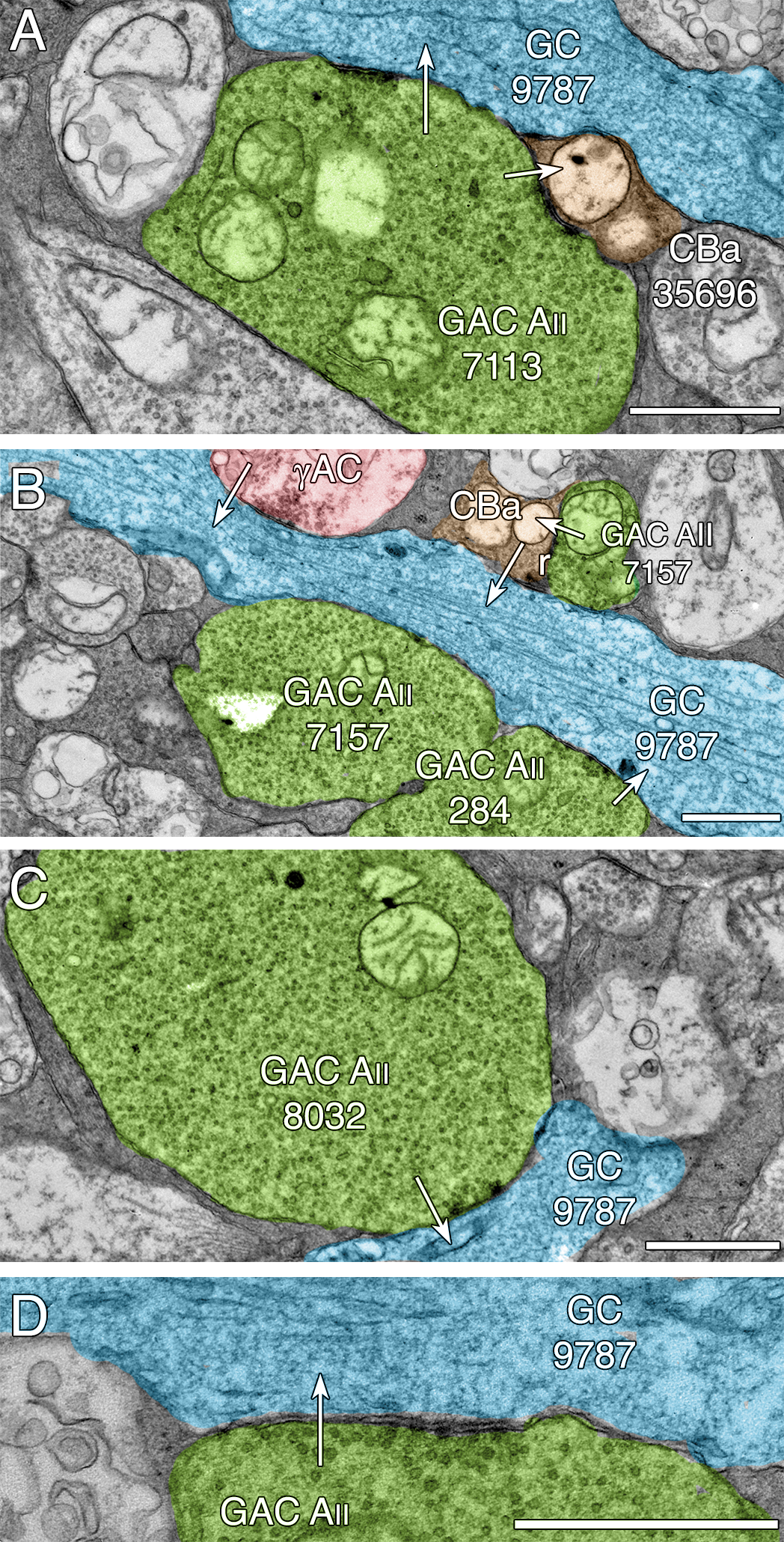

Supplement: FIGURE S2 — GC 9787 dendrites (cyan) collect multiple synaptic inputs from glycinergic AII amacrine cell distal lobular appendages (green) across the volume. (A) Conventional synapses from GAC AII 7113 onto both GC 9787 and CBa 35696 (tan) which is presynaptic to GC 9787 at two other sites. (B) Convergent signaling from γ+ amacrine cells (pink γ+ AC), GAC AII 284, and a CBa bipolar cell (tan) onto GC 9787. GAC AII 7157 makes synapses onto 9787 in another section (not shown) but is also presynaptic to the CBa bipolar cell. (C) Single synapse from lobule GAC AII 8032 onto GC 9787. (D) Classical multiple presynaptic densities associated with a single GAC AII synapse. Scales 1000 nm. [file Image_2.tif]
