# Supplementary material for: Heterocellular Coupling Between Amacrine Cells and Ganglion Cells
Source: Front Neural Circuits. 2018 Nov 14;12:90. doi: 10.3389/fncir.2018.00090 (PMC6247779; doi:10.3389/fncir.2018.00090)
Supplement: TABLE S1 — Examples of retinal cell classes, intermediate groups and superclasses. [file Table_1.pdf]

Table S1 Examples of retinal cell classes, intermediate groups and superclasses

Class	Intermediate Group	Intermediate Group	Superclass	Vertebrate Group
rods			photoreceptors	Mammalia
VP 502 rods		rods	photoreceptors	Amphibia
VP 432 rods		rods	photoreceptors	Amphibia
L cones		cones	photoreceptors	Mammalia
S cones		cones	photoreceptors	Mammalia
R long single cones	single cones	cones	photoreceptors	Cyprinidae, Teleosteii
G long single cones	single cones	cones	photoreceptors	Cyprinidae, Teleosteii
B short single cones	single cones	cones	photoreceptors	Cyprinidae, Teleosteii
R long double cone member	double cones	cones	photoreceptors	Cyprinidae, Teleosteii
G short double cone member	double cones	cones	photoreceptors	Cyprinidae, Teleosteii
rod BC			bipolar cells	Mammalia
CBb3 BC		cone bipolar cells	bipolar cells	Mammalia
Mb1 BC	ON bipolar cells	mixed rod-cone bipolar cells	bipolar cells	Cyprinidae, Teleosteii
Tristratified B cone BC	ON bipolar cells	pure cone bipolar cells	bipolar cells	Cyprinidae, Teleosteii
AII AC		Narrow field amacrine cell	amacrine cells	Mammalia
AI AC		Wide field amacrine cell	amacrine cells	Mammalia
Interstitial axonal cell (IAC)		Axonal cell*	amacrine cells	Mammalia
OFF α GC	transient GC	OFF ganglion cell	ganglion cell	Mammalia
ON transient DS GC	transient GC	ON ganglion cell	ganglion cell	Mammalia
ON β GC	sustained GC	ON ganglion cell	ganglion cell	Mammalia

* Interstitial axonal cells are also widely known as axon-bearing amacrine cells, an unfortunate construction meaning "axonless cells with axons." Described in rabbit by Famiglietti (1992) as polyaxonal or PA1 cells, we prefer the "axonal cell" grouping as a compact class descriptor.
