# Supplementary material for: Heterocellular Coupling Between Amacrine Cells and Ganglion Cells
Source: Front Neural Circuits. 2018 Nov 14;12:90. doi: 10.3389/fncir.2018.00090 (PMC6247779; doi:10.3389/fncir.2018.00090)
Supplement: TABLE S2 — GABA immunocytochemistry species list. [file Table_2.pdf]

Table 2. GABA immunocytochemistry species list

Superclass Cyclostomata

Class Myxina
Myxini sp. (hagfish)

Class Hyperoartia
Ichthyomyzon sp. (brook lamprey)
Petromyzon marinus (lamprey)

Class Chondrichthyes
Dasyatis sabina (ray)
Hydrolagus collei (ratfish)
Raja spp. (skates)
Squalus acanthias (shark)
Mustelus canis (shark)

Superclass Osteichthyes
Protopterus sp. (lungfish)
Calamoichthyes sp. (reedfish)
Polypterus palmas (bichir)
Lepisosteus osseus (gar)
Carassius carassius (carp)
Carassius auratus (goldfish)
Ictalurus punctatus (catfish)
Lepomis sp. (sunfish)
Micropterus sp. (bass) 0
Lumpenis saggita (snakefish)
Porichthyes notatus (midshipman)
Cymatogaster aggregata (shiner perch)
Betta splendens (betta)
Mormytus sp. (elephantfish)

Class Amphibia
Lithobates spp. (frogs)
Triturus viridescens (salamander)
Salamandra salamandra (salamander)
Pleurodeles waltli (salamander)
Ichthyophis kohtaoensis (gymnophionid)
Xenopus laevis (clawed frog)
Bufo marinus (toad)
Necturus maculosus (mudpuppy)
Ambystoma tigrinum (tiger salamander)
Amphiuma means (congo eel)
(Golden newt)

Class Reptilia
Anolis carolinensis (anolis)
Trachemys scripta elegans (turtle)
Xantusia vigilis (night lizard)
Thamnophis sirtalis (garter snake)
Lampropeltis sp. (King snake)

Class Aves
Gallus domesticus (chicken)
Columba livia (pigeon)

Class Mammalia
Setonix brachyurus (wallaby)
Rattus norvegicus (rat)
Mus musculus (mouse)
Oryctolagus cuniculus (rabbit)
Sus domesticus (pig)
Odocoileus sp. (American deer)
Cavia porcellus (guinea pig)
Capra aegagrus hircus (goat)
Papio anubis (baboon)
Saimiri sciureus (squirrel monkey)
Homo sapiens (human)
Macaca spp. (macaques)
Felis silvestris catus (cat)
Canis lupus familiaris (dog)
Mustela putorius furo (ferret)
