# Supplementary material for: Heterocellular Coupling Between Amacrine Cells and Ganglion Cells
Source: Front Neural Circuits. 2018 Nov 14;12:90. doi: 10.3389/fncir.2018.00090 (PMC6247779; doi:10.3389/fncir.2018.00090)
Supplement: TABLE S3 — Log10 relative ligand required to block tissue binding. [file Table_3.pdf]

Table S3: Log_{10} relative ligand required to block tissue binding

	IgG							
**Ligand**	**A**	**D**	**E**	**G**	**J**	**Q**	**τ**	**γ**
**A-GA-A**	-	9	5	7	9	7	6	5
**D-GA-D**	7	-	5	10	9	7	6	6
**E-GA-E**	5	7	-	9	9	5	6	4
**G-GA-G**	3	8	5	-	9	7	6	6
**J-GA-J**	7	9	4	9	-	6	5	5
**Q-GA-Q**	4	9	5	9	9	-	6	6
**τ-GA-τ**	5	8	5	10	9	7	-	5
**γ-GA-γ**	2	10	5	8	9	7	6	-

Legend. Top row, IgG targets; left column, binding competitor. A alanine, D L-aspartate, E L-glutamate, G glycine, J glutathione, Q L-glutamine, τ taurine, γ GABA. Each IgG was used at the optimal dilution for quantitative detection (previously determined from saturation assays) on a standard ultrathin section of retinal tissue used to calibrate all IgGs. Dilutions: IgG A (1:10000), IgG D (1:2000), IgG E (1:32000), IgG G (1:4000), IgG J (1:4000), IgG Q (1:2000), IgG τ (1:16000), IgG γ (1:32000). Competitors were created by adding 2 moles of free amine / mole of purified glutaraldehyde in a pH 7.4 0.1M phosphate buffer medium, resulting in a nominal bis-amine ligand mimicking 2 molecules of tissue antigen/molecule competitor. Competitors were applied over a 12 log_{10} unit range with IgGs to standard samples, visualized with CMP, and the concentration required for 100% blockade if IgG binding to known cellular targets determined for each IgG-competitor pair. The absolute blocking concentrations for the cognate pairs (e.g. IgG A vs A-GA-A) were: A 1 nM, D 1 pM, E 100 nM, G 1 pM, J 10 pM, Q 1 nM, τ 10 nM, γ 10 nM. The cognate values were set to 0 and the differentials with other pairs tabulated. For example, the competitor J-A-J displaces IgG J 10^9-fold more effectively than any other ligand. For tissue level detection, this constitutes virtually absolute selectivity: a 5 mM tissue level of glutathione generates a high level of IgG J binding and very strong signal in cells. To generate any contamination of that signal, glutamate or any other competitor would have to be present at physiologically unreachable levels. Similar arguments hold for the entire set.
